# Edge-assisted adaptive offloading algorithm for 3D object detection tasks

**DOI:** 10.1371/journal.pone.0345876

**Published:** 2026-04-16

**Authors:** Kangli Zhao, Zhongrui Gou, Huaqing Liu

**Affiliations:** 1 School of Computer Science and Technology, Aba Teachers University, Aba, Sichuan, China; 2 School of Computing and Artificial Intelligence, Southwest Jiaotong University, Chengdu, Sichuan, China; Van Lang University: Truong Dai hoc Van Lang, VIET NAM

## Abstract

Multimodal 3D object detection is crucial for autonomous systems but suffers from high delay due to significant computational demands. To address this, we propose an edge computing-assisted framework that balances load between terminal devices and edge servers. We introduce dynamic threshold tuning and resolution-adaptive offloading algorithms to optimize performance. Experimental results demonstrate that our approach significantly reduces delay by minimizing offloading frequency while maintaining high accuracy, achieving a superior delay-accuracy trade-off. Furthermore, the framework exhibits robust adaptability across various models and bandwidth conditions, ensuring effectiveness in dynamic environments.

## Section 1: Introduction

In recent years, the number of autonomous vehicles has been steadily increasing, with projections indicating that by 2030, the global fleet of autonomous vehicles will reach 2.2 million [1]. Autonomous driving has brought new industry development opportunities to the traditional automotive sector. Its diversified application scenarios, operational efficiency, and immense development potential have led many countries to view it as one of the key technologies for improving urban operations and creating economic value. For instance, the UK government announced its Connected and Automated Mobility (CAM) Roadmap in 2022, aiming to promote the adoption of autonomous vehicles by 2025 [2]. The U.S. Department of Transportation plans to fully implement cellular-based V2X (Vehicle-to-Everything) coverage by 2036 to further support higher levels of autonomous driving services [3]. Japan plans to achieve a digital transformation in the automotive sector with software-defined vehicles between 2030 and 2035 [4].

Autonomous driving relies on various sensors and algorithms to quickly perceive the environment and make rapid decisions, ensuring the timeliness and reliability of driving decisions. 3D object detection, which collects data through sensors such as LiDAR or RGB-D cameras, enables object recognition and localization in 3D space [5]. It has become one of the most commonly used object recognition technologies in the field of autonomous driving. Algorithms allow the vehicle to quickly understand environmental data, enabling it to avoid potential hazards [6]. In addition, point cloud object detection plays an irreplaceable role in perceiving the three-dimensional world, driving advancements across diverse fields. In autonomous driving, it supports critical tasks such as pavement data acquisition and analytics, enhancing vehicle safety and infrastructure monitoring [7]. Beyond automotive applications, it is essential in intelligent robotics, enabling precise object identification on platforms like Nao robots and facilitating multimodal detection using depth and image data for manufacturing parts [8]. Furthermore, these detection technologies synergize with Internet of Things (IoT) frameworks to support cooperative models for emergency transportation planning. To ensure efficient deployment in these complex scenarios, optimal inferential control of convolutional neural networks is often employed to balance performance and computational cost. Collectively, these applications demonstrate the broad adaptability and significance of 3D object detection in modern intelligent systems.

Multimodal 3D object detection technology fully combines the advantages of deep learning and multimodal data, significantly improving the model’s accuracy [9] and overcoming the inherent limitations of single-modal detection in acquiring depth information. However, this method requires significant computational resources. Given the limited computational power of in-vehicle terminal devices, requiring them to execute all object detection tasks leads to a decline in inference speed and accuracy. Furthermore, it fails to meet the stringent delay requirements for task processing in autonomous driving. Meanwhile, LiDAR supports a point cloud sampling rate of up to 8 million points per second, which, in turn, leads to a significant increase in inference delay due to the high-speed point cloud sampling rate [10]. To improve the processing efficiency of 3D point cloud data, some studies [11–12] have employed lightweight model techniques, such as knowledge distillation or neural network pruning, to accelerate the inference process. However, lightweight models may not provide sufficient performance to support complex learning tasks and may introduce additional communication and computational overhead for retraining [13]. Connected and Autonomous Vehicles (CAVs) [14] can leverage cellular vehicle-to-everything communication (C-V2X) [15], vehicle-to-infrastructure communication (V2I), and multi-access edge computing (MEC) [16] to enable data exchange and task transmission between vehicles, edge nodes, and cloud servers. This facilitates the acquisition of complementary environmental information, the allocation of computational resources, and provides a solid technological foundation for the application of edge computing in vehicular scenarios. The vehicular edge computing paradigm [17] allocates computational and storage resources to network edge servers and deploys multimodal deep learning models. By offloading part or all of the tasks from in-vehicle terminals to edge servers for proximal processing, it improves task computational efficiency. However, 3D object detection tasks based on edge computing still face several technical challenges, such as the significant bandwidth consumption required for transmitting multimodal data, which leads to severe delay issues.

Designing efficient and energy-saving 3D object detection models is critical for ensuring the safety of autonomous driving [7,18]. Due to the large and complex nature of point cloud data, which demands high computational power, edge computing has become an ideal computing paradigm for rapidly addressing 3D object detection tasks. Considering that information exchange between endpoints can enhance both the accuracy and speed of object detection, several scholars [6,19,20] have thoroughly explored the design of end-to-end frameworks, capturing correlations between tracked objects and facilitating the interaction of computational tasks. Additionally, given the limited resources in edge computing environments, some studies have focused on optimizing the computation offloading mechanisms to improve the efficiency of collaborative edge-based object detection systems. Parameters such as frame resolution, frame rate, and confidence thresholds [21–22] are now being utilized to enable adaptive task offloading decisions, aimed at meeting the performance requirements of dynamic scenarios, including accuracy, throughput [23], and energy consumption [24].

However, current edge-assisted object detection technologies are primarily based on image data, with insufficient research on point cloud data generated by LiDAR. In fact, point cloud object detection plays an irreplaceable role in perceiving the three-dimensional world, driving the advancement and application of autonomous driving, intelligent robotics, virtual gaming, and other fields [25–26]. Unlike image data, the quality of point cloud data is characterized by the point cloud resolution parameter, and the relationship between this resolution and accuracy and delay is still unclear. Therefore, existing offloading mechanisms cannot be directly applied to 3D object detection scenarios.

**Fig 1:**
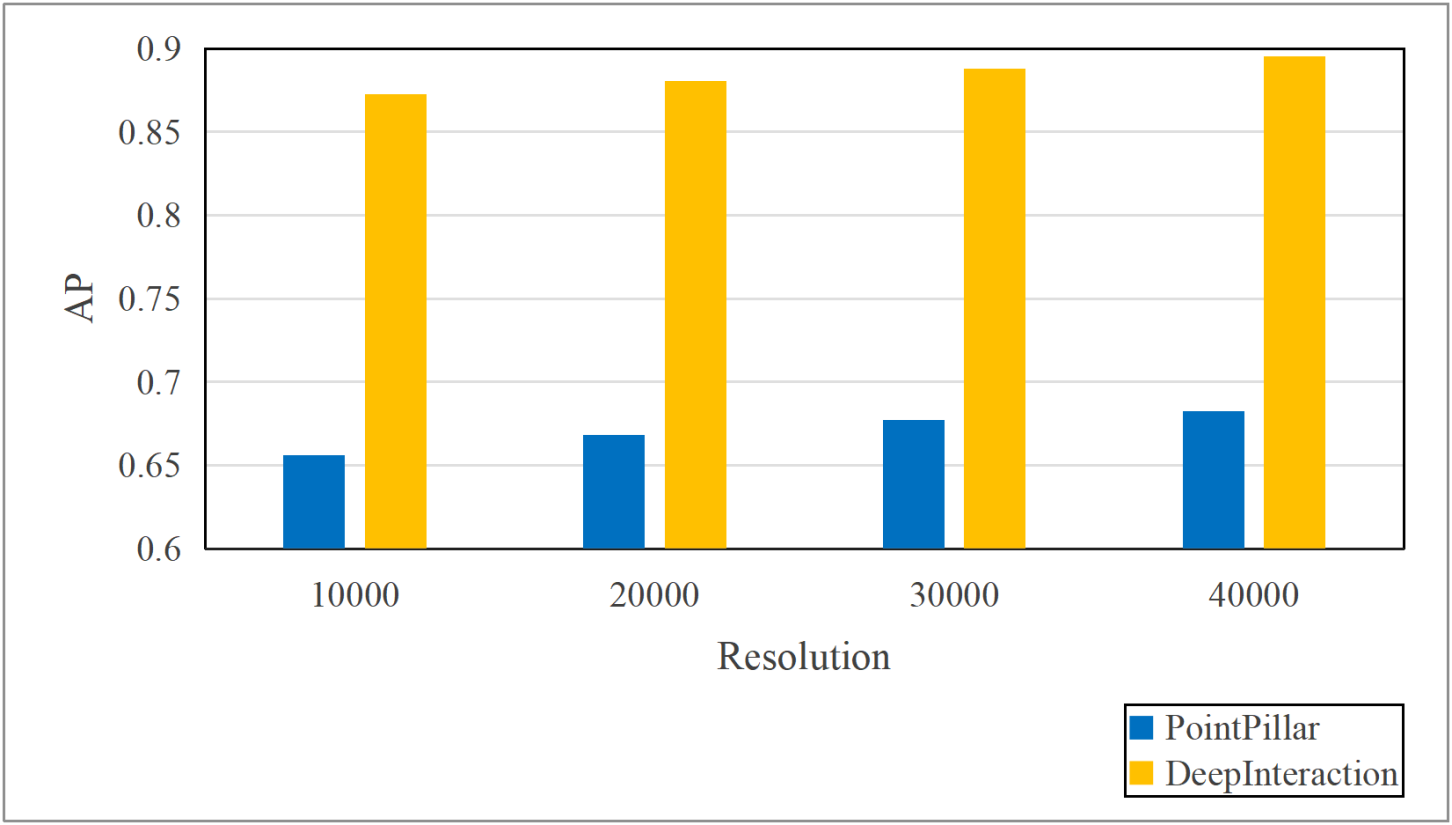
AP comparisons of single-modal and multimodal models at different point cloud resolutions.

**Fig 2:**
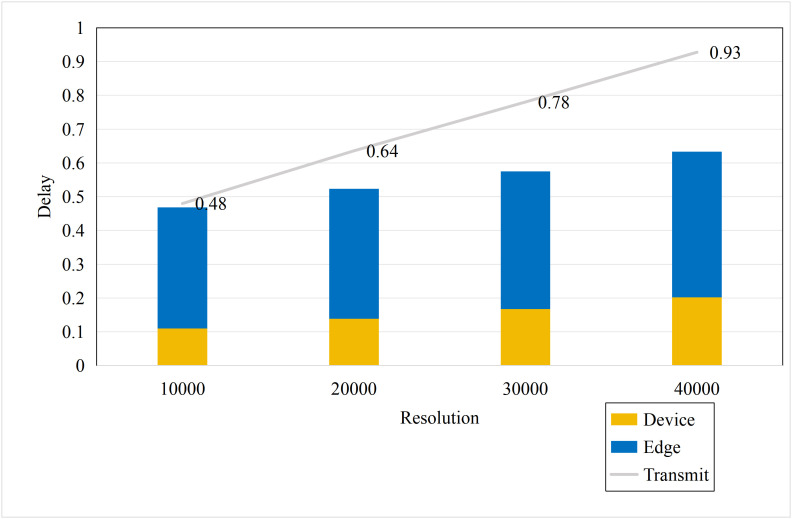
Delay comparisons of single-modal and multimodal models at different point cloud resolutions.

Based on the aforementioned motivations, this paper proposed a highly efficient computational algorithm to quantify the data upload priority of different modalities. Furthermore, our proposed framework enhances computational efficiency by dynamically distributing the inference workload. The lightweight terminal model filters out easy samples, reducing the frequency of expensive edge inferences, while the adaptive resolution mechanism minimizes the data volume for necessary offloading, optimizing both computational and communication resources. Experimental results show that point cloud resolution and confidence significantly influence the performance of 3D object detection. On the one hand, point cloud resolution, defined as the number of points per unit volume, characterizes the fine-grained details of the point cloud. Fig 1 illustrates the inference performance of the unimodal model PointPillar [27] and the multimodal model DeepInteraction [28] under different point cloud resolutions. In this study, “Device” refers to the efficiency of the point cloud-based unimodal detection model (PointPillar) deployed on local devices. “Edge” denotes the efficiency of the image-point cloud-based multimodal detection model (DeepInteraction) deployed on edge devices. Furthermore, “Transmit” signifies the delay associated with data offloading and transmission to edge devices during multimodal data inference based on edge collaboration. As shown in Fig 2, the point cloud resolution increases from 1 × 10^4^ pts/m³to 4 × 10^4^ pts/m³, the inference delay of the unimodal model rises by 0.1 seconds, while the multimodal inference delay increases by 0.07 seconds, and the transmission delay increases by 0.4 seconds. Fig 1 and Fig 2 illustrate that with higher point cloud resolutions, both the accuracy of the unimodal and multimodal models improves; however, this also leads to significant increase in transmission delay and inference. Therefore, considering the impact of point cloud resolution is essential for optimizing the edge-device collaborative 3D object detection model. The experimental setup detailed in Section 4.

Image resolution is represented by pixel density, while point cloud resolution reflects the density and distribution of points in 3D space. To assess the effect of image resolution on detection performance, we varied the image resolution and observed the corresponding changes in accuracy, as summarized in Table 1. As illustrated in Table 1, reducing the image resolution from the original 1600 × 900–400 × 300 results in a decrease of Average Precision (AP) by less than 0.01, indicating that image resolution has a minimal impact on accuracy.. Instead, we select point cloud resolution and confidence threshold as the key metrics. The confidence threshold serves as the decision trigger: when the confidence of local detection falls below this threshold, the terminal offloads the multimodal data to the edge server for higher-precision inference. However, due to the high-speed movement of vehicles, network conditions change rapidly, and traffic patterns become unpredictable, posing challenges in designing effective task offloading mechanisms for edge-assisted 3D object detection frameworks [29–30]: The transmission delay of point cloud data with the same resolution varies with the network environment, the confidence of object detection changes dynamically with the detection content, and a low confidence threshold can lead to frequent offloading, causing excessive transmission delay.

**Table 1:** The value of AP corresponding to different image resolutions.

Resolution (pixels)	400 × 300	800 × 450	1280 × 720	1600 × 900
**AP**	0.902932	0.905026	0.908691	0.911309

To address the above challenges, the main contributions of this paper are summarized as follows:

- This paper proposes an edge-assisted 3D object detection framework that leverages the heterogeneous resource characteristics of terminal devices and edge servers to enable collaborative inference of unimodal and multimodal detection models.
- A detailed analysis is provided on the impact of point cloud resolution and confidence threshold on the delay and accuracy of object detection. Based on this, a mathematical optimization problem is formulated to minimize delay while meeting accuracy requirements.
- A comprehensive offloading algorithm is proposed in this paper, which includes accuracy estimation, threshold updating, and resolution adjustment. To evaluate accuracy in real-time, a confidence-based accuracy estimation method is designed, and a confidence-accuracy mapping relationship is established using quadratic fitting functions. Based on this, dynamic threshold updating and adaptive offloading algorithms are proposed, which fine-tune the threshold based on accuracy estimated from the previous cycle and dynamically adjust the resolution according to real-time bandwidth to balance accuracy and delay.
- An edge-terminal collaborative hardware experimental platform is introduced, which unimodal and multimodal object detection models are implemented on the terminal device and edge server, respectively. The performance of the models is evaluated using the nuScenes [31] and KITTI [32] datasets. Experimental results demonstrate that, compared with five baseline algorithms, the proposed approach achieves optimal overall performance in terms of both delay and accuracy under various testing scenarios.

The remainder of this paper is organized as follows: Section 2 introduces related work. Sections 3 describes the system model and algorithm design. Section 4 discusses the experimental platform and verifies the effectiveness of the proposed algorithm. Finally, Section 5 concludes the paper.

## Section 2: Related works

Autonomous vehicles, intelligent robots, and logistics systems depend on multi-sensor perception to make informed decisions for safety and efficiency [33–34]. While deep learning has significantly advanced 3D object detection, particularly through one-stage methods like PointNet [35] and PointGNN [36], and two-stage approaches like PointPillars [27], achieving a balance between accuracy and real-time performance remains a challenge. Advanced methods often rely on complex operations such as sparse 3D convolutions or transformers [13], which demand substantial computational resources. Although these techniques achieve high accuracy in experimental settings, their high parameter counts and computational overhead frequently create inference bottlenecks, making them difficult to deploy on resource-constrained terminal devices where real-time responsiveness is critical [37–38].

In the realm of multimodal data fusion, edge-cloud collaborative frameworks typically employ early, intermediate, or late fusion strategies. Early fusion [39–40] optimizes data quality but consumes excessive bandwidth, while late fusion [41] minimizes bandwidth usage but often sacrifices accuracy by ignoring intrinsic inter-modal correlations. Intermediate fusion [6,42] offers a promising middle ground by balancing accuracy and delay through feature-level transmission. However, research in this area remains insufficient; most existing studies focus solely on model feature extraction without adequately addressing the implications of rapidly changing communication environments. Furthermore, current approaches often prioritize average accuracy metrics while neglecting critical performance indicators such as end-to-end delay [43–44], which are essential for safety-critical applications.

From the perspective of application scenarios, existing methodologies often lack the environmental adaptability required for real-world deployment. First, many methods depend on stable, high-quality network environments and struggle to cope with significant fluctuations or signal interruptions, leading to unacceptable delay spikes [45]. Second, current collaborative frameworks typically rely on rigid task partitioning that makes terminals dependent on edge servers for inference; if the edge becomes unavailable or overloaded, the terminal cannot operate independently [39,46]. Finally, existing works primarily optimize model structures for theoretical accuracy, ignoring the impact of external environmental factors—such as dynamic bandwidth availability and computing resource contention—on system performance. Consequently, these methods are often ill-suited for the dynamic and variable environments characteristic of autonomous driving and intelligent robotics. Table 2 presents the similarities and differences between our study and existing studies.

**Table 2:** Difference between our scheme and the most relevant schemes.

Ref.	Bandwidth	Data Quality	End-to-End delay	Dynamic Environment	Terminal Device Independence
[6]	√	√	×	×	√
[39]	√	×	×	×	√
[40]	√	×	×	×	×
[41]	√	√	×	×	√
[42]	√	√	×	√	×
[43]	×	√	×	×	√
[44]	×	√	×	×	√
[45]	√	√	×	√	√
[46]	√	√	√	√	×
Ours	√	√	√	√	√

The aforementioned research effectively leverages the heterogeneous resources of terminal devices, achieving a reduction in the transmission volume of multimodal data while maintaining accuracy. Nevertheless, certain areas still necessitate urgent enhancements:

(1) Existing methods rely on a high-quality network environment to achieve real-time data transmission, which makes them unable to cope with scenarios involving significant network fluctuations, signal interruptions, etc. This results in extremely high delay, making them unsuitable for applications that require very low delay.
(2) The existing edge-collaboration frameworks are based on task or model partitioning, requiring the terminal to rely on the edge for subsequent model inference. This dependency limits the terminal’s ability to operate independently and may result in increased delay.
(3) Current approaches primarily focus on designing collaboration strategies that consider the structural characteristics of the model in relation to accuracy, while neglecting the inherent connection between external environmental factors and model performance. For instance, fluctuations in bandwidth and the availability of computational resources are often overlooked, rendering these frameworks inadequately equipped to adapt to the dynamic and fluctuating nature of real-time environments.

In contrast to the aforementioned studies, this paper proposes a 3D object detection framework based on the collaboration of single-modal and multimodal models. In this framework, lightweight single-modal models and computationally intensive multimodal models are deployed at resource-constrained terminals and edge servers with higher computational capacity, respectively. On the terminal side, the lightweight single-modal model, with its high efficiency, can quickly respond to and process local data, providing preliminary and reliable prediction results for subsequent multimodal inference. This design significantly reduces dependence on network bandwidth while alleviating the computational burden on edge servers. On the edge side, the complex multimodal model can integrate various data sources to perform in-depth feature extraction and fusion, further improving the accuracy and robustness of object detection. Additionally, this paper delves into the impact of several key variables on delay and accuracy. Through a series of experimental results, the inherent relationships between these variables are analyzed, providing a theoretical basis for offloading decision-making. Based on the aforementioned research, a lightweight offloading algorithm is designed to dynamically adjust key parameters such as confidence thresholds and real-time resolution, based on real-time predictions of model accuracy, in order to adaptively balance delay and accuracy in real-time environments, thus meeting the diverse demands of various application scenarios.

## Section 3: System model and algorithm design

### 3.1. System model

In this section, a framework for a 3D object detection system based on edge-assisted technology is proposed, consisting primarily of terminal devices and edge servers. In contrast to existing edge-assisted architectures, the proposed framework introduces three key innovations: (1) A heterogeneous model deployment strategy that pairs a lightweight single-modal model on the terminal with a complex multimodal model on the edge to optimize resource utilization; (2) A dynamic confidence threshold update mechanism that adapts to real-time accuracy requirements, acting as an intelligent gatekeeper for offloading; and (3) An adaptive resolution selection algorithm that minimizes transmission delay by optimizing data volume based on current network bandwidth and delay constraints. These components work synergistically to balance accuracy and efficiency in dynamic environments.

**Fig 3:**
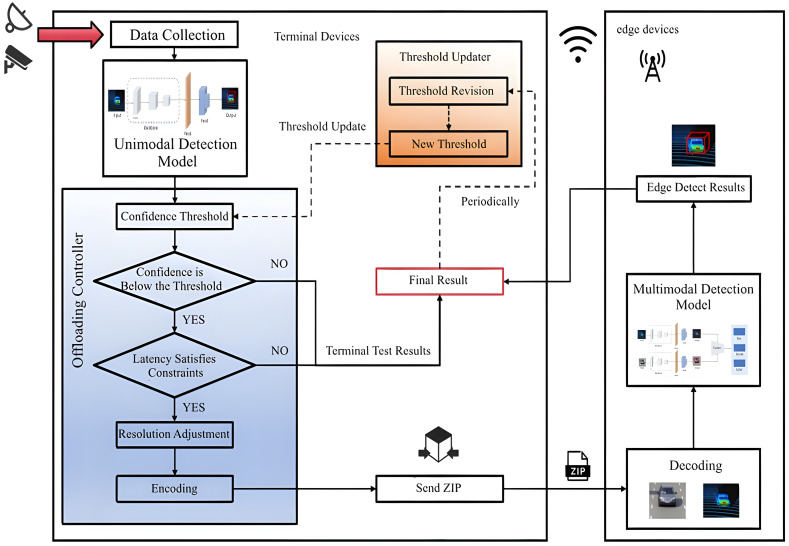
Edge-assist framework of 3D object detection.

As shown in Fig 3, the terminal device is composed of four modules: data collection, local inference, offloading controller, and parameter configuration updater. First, the data collection module is responsible for acquiring multimodal data, including video and point cloud data. In autonomous driving scenarios, the terminal device is typically a mobile vehicle equipped with various sensors, including onboard cameras and LiDAR. These sensors collect image and point cloud data at a fixed sampling frequency. The local inference module deploys a single-modal inference model, PointPillar, based on point cloud data. We selected PointPillar specifically for its lightweight architecture and low parameter count, enabling efficient real-time execution on resource-limited terminal hardware while maintaining sufficient accuracy for initial filtering. This module processes the point cloud data and provides the results and confidence levels for the detection tasks. The offloading controller makes decisions regarding the offloading of detection tasks and the point cloud resolution based on the confidence threshold and accuracy requirements. The point cloud resolution is defined as the number of points divided by the volume of the object, which determines the density of the point cloud data and influences the size of the point cloud data to be transmitted during the offloading process.

**Fig 4:**
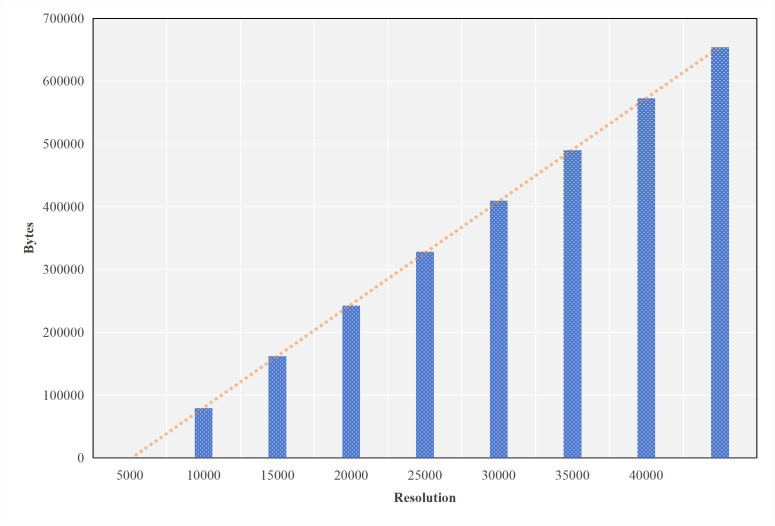
Data size of different point cloud resolutions.

As shown in Fig 4, the size of the point cloud data is linearly correlated with its resolution. When the confidence level of the local inference result does not meet the threshold, multimodal data is offloaded to the server for further inference. The threshold updater is responsible for dynamically updating the confidence threshold. On the edge server side, a multimodal detection model is deployed to assist the terminal device with inference, thereby improving detection accuracy.

**Fig 5:**
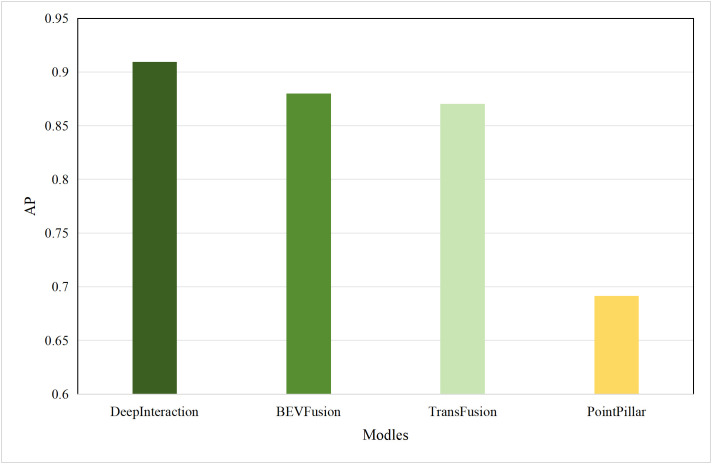
AP values for single-modal and multimodal models.

**Fig 6:**
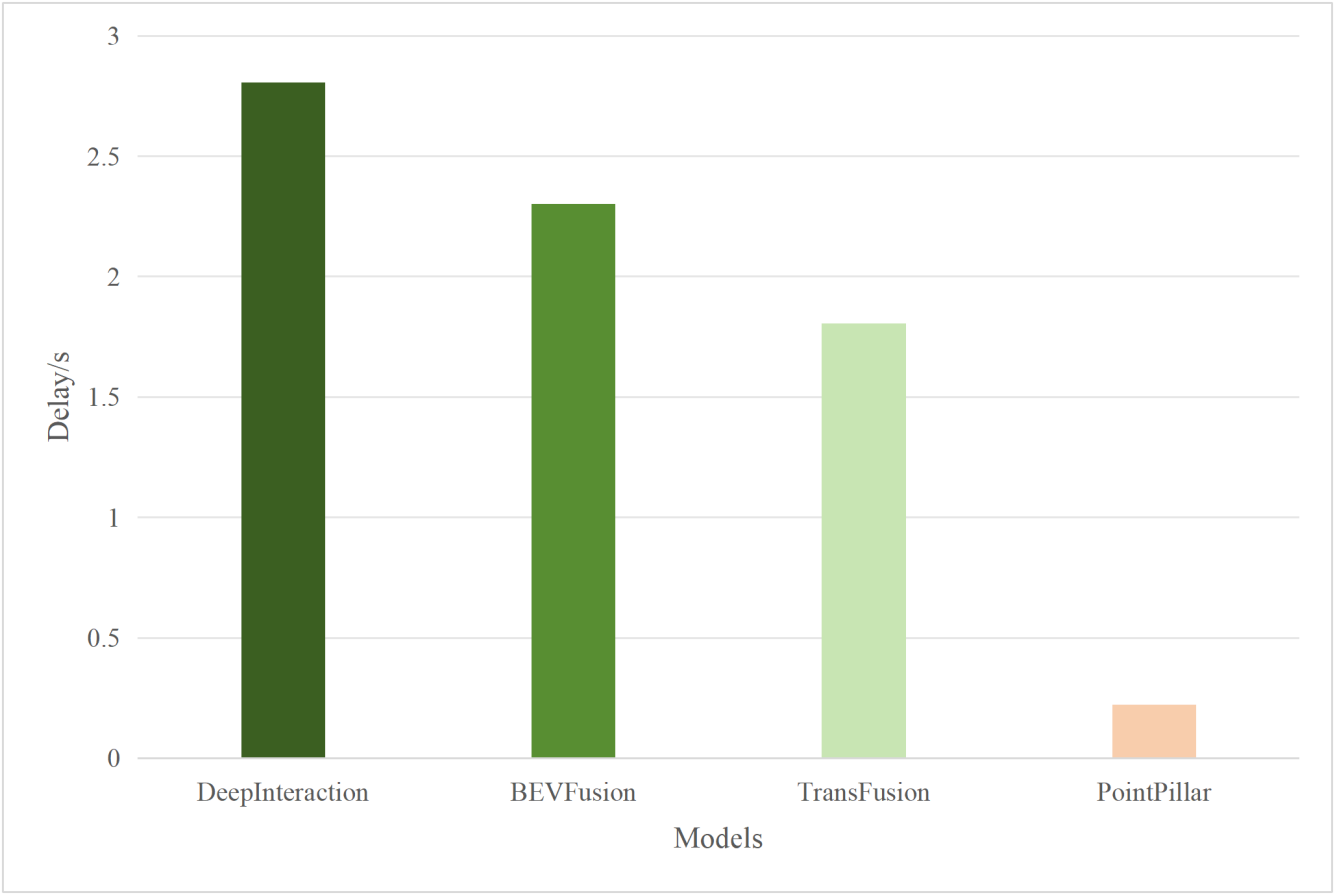
Delay values for single-modal and multimodal models.

**Fig 7:**
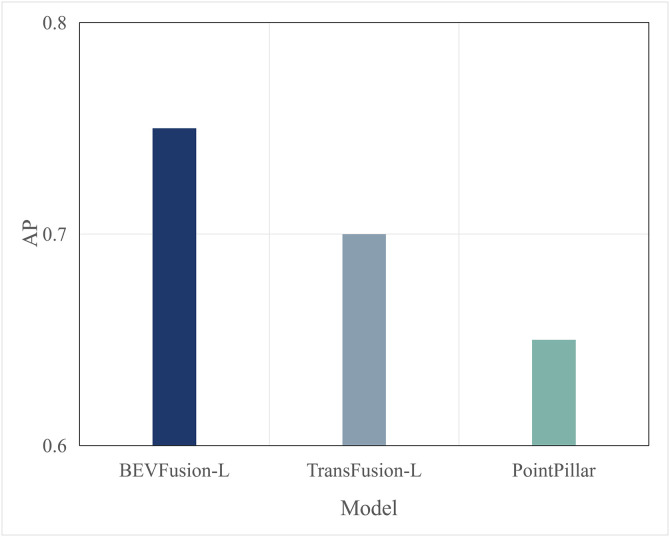
AP values of single-modal.

**Fig 8:**
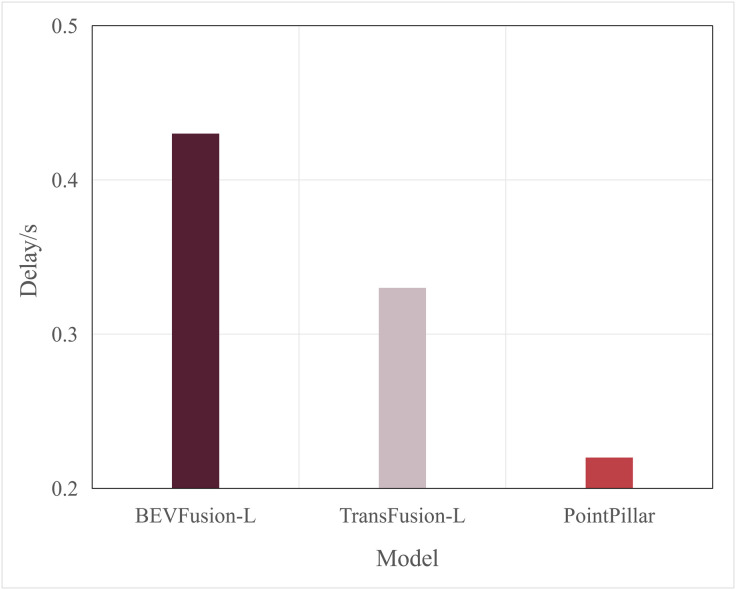
Delay values of single-modal.

Typically, multimodal models provide better accuracy than single-modal models. Fig 5 and Fig 6 present performance comparisons between single-modal and multimodal inference models on terminal devices, with the terminal device being the NVIDIA Jetson AGX Xavier. It is evident that the accuracy of the three multimodal models, DeepInteraction, Transfusion [47], and BEVFusion [48], surpasses that of the single-modal model, PointPillar, with significantly higher inference delay as well. Additionally, Fig 7 and Fig 8 compare the performances of various single-modal models on the terminal device. Although PointPillar has the lowest accuracy among the three single-modal models, it also exhibits the lowest inference delay. Moreover, under the proposed offloading strategy, higher-accuracy single-modal models reduce offloading frequency by correctly classifying more samples locally. As a result, using these models on the terminal does not necessarily outperform PointPillar when considering the overall balance between accuracy and latency. Consequently, to balance delay and accuracy, PointPillar is chosen as the inference model for the terminal device. Furthermore, DeepInteraction, which has the highest inference accuracy, is used as the inference model on the edge side to compensate for the lower accuracy of terminal model inference in certain scenarios.

Next, the specific processing flow of the proposed edge-assisted collaborative object detection framework is described as follows and shown in Fig 3. In the first step, the data collection module gathers multimodal data through sensor data; In the second step, the local inference model performs single-modal object detection on the collected point cloud data to obtain the detection confidence of the terminal device. In the third step, based on the terminal’s detection confidence, the offloading controller makes the offloading decision. If the confidence level exceeds the preset threshold, the terminal’s detection result is used as the final result. Otherwise, based on real-time bandwidth and accuracy requirements, the point cloud resolution is dynamically adjusted, and the data, along with image data, is compressed and sent to the terminal device for further processing. In the fourth step, the terminal device receives the compressed data packet, decodes it, and processes it with the multimodal model, returning the result to the terminal. During this process, the threshold updater will collect and analyze the results and periodically update the confidence threshold based on the established update strategy. The threshold update strategy will be described in detail in Section 4 of the algorithm.

The overall computational complexity analysis of the proposed edge-assisted adaptive offloading algorithm is as follows:

Offloading decision module: The offloading decision for each task involves evaluating the local inference cost and the edge inference cost (including transmission delay). For *N* tasks and *K* candidate edge servers, the complexity of the offloading decision process is O(NK).

Point cloud resolution adaptation module: The resolution selection involves searching over *R* candidate resolution levels to optimize the accuracy-delay trade-off, contributing a complexity of O(R) per task.

Overall complexity: The total complexity of the proposed algorithm is O(NK+NR). Since *K* and *R* are typically small constants in practical deployment scenarios, the algorithm scales linearly with the number of tasks *N,* ensuring computational efficiency suitable for real-time applications.

### 3.2. Problem description

In this section, a detailed analysis of the above-mentioned offloading process is conducted, and a theoretical optimization model is established. The definitions of the symbols used in this article are shown in Table 3.

**Table 3:** Main symbols and definitions.

Symbol	Definitions
R	the set of selectable resolutions data
$A_{req}$	the required accuracy
mAP	the average accuracy
$D=\left\{d_{n}\right\}$	dataset
$\overset{d}{\underset{n}{c}}$	confidence of sample $d_{n}$
$\overset{e}{\underset{n}{c}}(r)$	edge confidence of the sample $d_{n}$ with resolution r
$\overset{d}{\underset{n}{A}}$	accuracy of sample $d_{n}$
$\overset{e}{\underset{n}{A}}$	accuracy of terminal device model
$t_{n}(r)$	the processing delay of sample$d_{n}$ with resolution r
$\overset{e}{\underset{n}{t}}(r)$	inference delay at the edge server of sample$d_{n}$ with resolution r
$\overset{t}{\underset{n}{t}}(r)$	the transmission delay of sample$d_{n}$ with resolution r
r	the resolution of sample$d_{n}$
$T_{max}$	the maximum allowable delay
$D_{r}$	the threshold increment
ρ	confidence threshold
Δρ	confidence threshold adjustment step
λ	scale factor

When the Sample $d_{n}$ is processed by the local model at the original resolution $r_{max}$, the processing delay is equal to the inference delay of the local model, as shown in equation (1):


$$t_{n}=\overset{d}{\underset{n}{t}}\;,ifd_{n}isprocessedbythelocalmodel$$ (1)

Equation (2) indicates that when a sample $d_{n}$ with resolution r is offloaded to the edge server, its processing delay is the sum of the transmission delay and the inference delay at the edge server.


$$T_{n}=\overset{t}{\underset{n}{t}}(r)+\overset{e}{\underset{n}{t}}(r)$$ (2)

where $\overset{t}{\underset{n}{t}}(r)$ represents the transmission delay for sample $d_{n}$, calculated as the sample size divided by the transmission rate, as shown in below:


$$\overset{t}{\underset{n}{t}}(r)=\frac{a\times r}{B}$$ (3)

Here a is a proportional coefficient used to calculate the size of the point cloud data, and B is the real-time transmission rate, which dynamically changes based on network conditions. $\overset{e}{\underset{n}{t}}(r)$ is the inference delay at the edge server for the sample $d_{n}$ with resolution r.

To model the computational cost efficiently, we analyzed the relationship between inference delay and point cloud resolution. As illustrated in Fig 2, extensive empirical tests on the NVIDIA GeForce RTX 3090 using the BEVFusion model reveal a strong linear correlation between resolution and inference delay. Consequently, we adopt a linear model for real-time delay estimation. This approximation significantly reduces optimization complexity while maintaining sufficient accuracy for the offloading decision. Thus, $\overset{e}{\underset{n}{t}}(r)$ exhibits a linear relationship with the resolution r, i.e., $\overset{e}{\underset{n}{t}}(r)=b_{\text{1}}\times r+b_{\text{2}}$, where the parameters $b_{\text{1}}$ and $b_{\text{2}}$ can be obtained through curve fitting.

Based on the above, the problem addressed in this paper is defined as follows: given a multimodal data set $D=\left\{d_{\text{1}},d_{\text{2}},\ldots ,d_{N}\right\}$, a set of selectable point cloud resolutions R, and a target accuracy requirement $A_{req}$, the objective of this problem is to minimize the total system delay by optimizing the point cloud resolution r and the confidence threshold ρ.the specific mathematical expression is as follows:


$$\underset{r,\rho }{\text{min}}\sum_{n=\text{1}}^{N}t_{n}$$ (4)

Subject to:


$$C_{\text{1}}:t_{n}\leq T_{max},n=\text{1},\text{2},\ldots ,N$$

$$C_{\text{2}}:mAP\geq A_{req}$$

where constraint $C_{\text{1}}$ ensures that the processing delay for each data frame does not exceed the maximum allowable delay $T_{max}$, and constraint $C_{\text{2}}$ ensures that the mAP is no less than the given accuracy requirement $A_{req}$, since *r* is a discrete variable and ρ is a continuous variable, this model constitutes a linear mixed-integer optimization problem, which is difficult to solve optimally in polynomial time.

**Fig 9:**
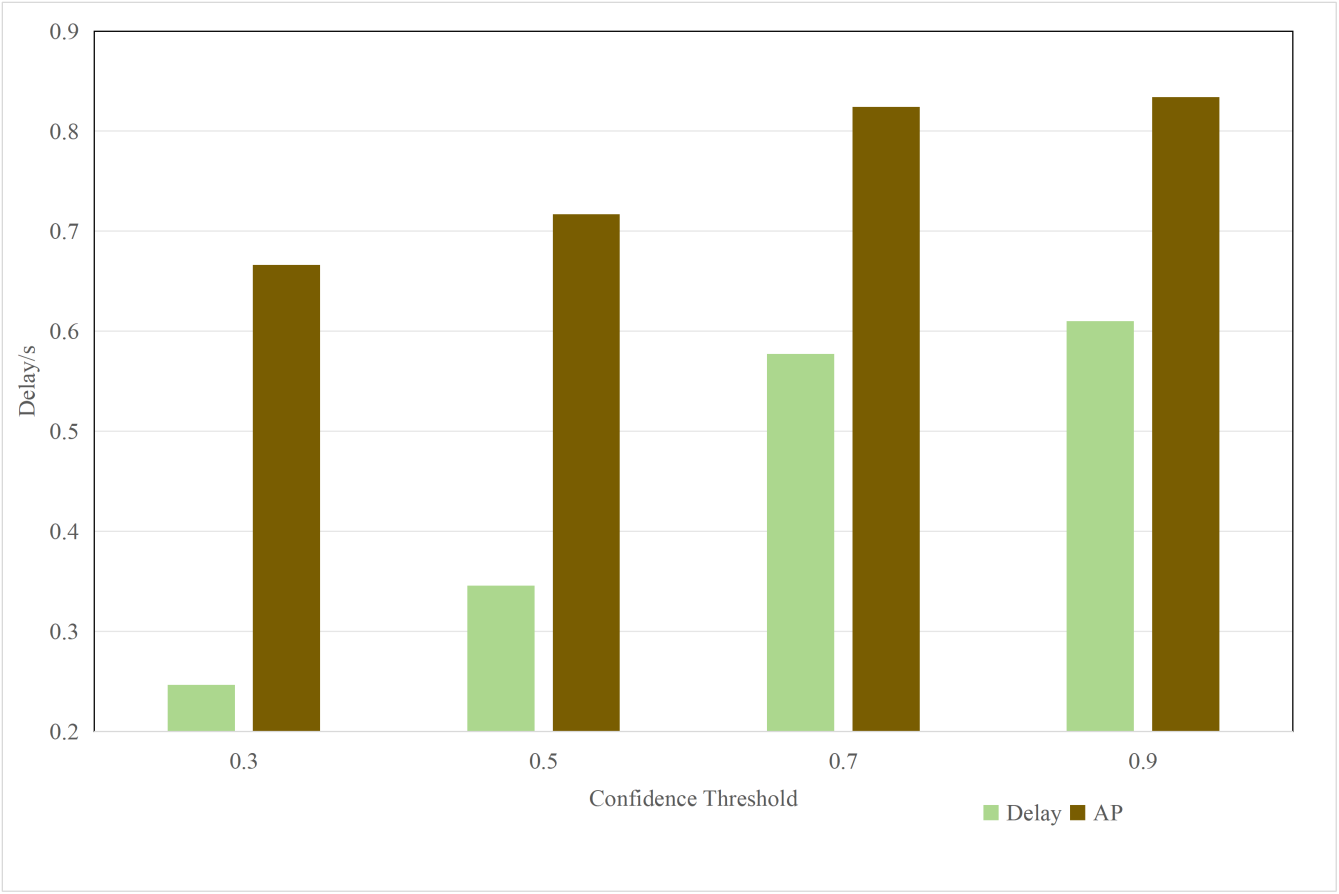
Performance comparison under different confidence thresholds.

**Fig 10:**
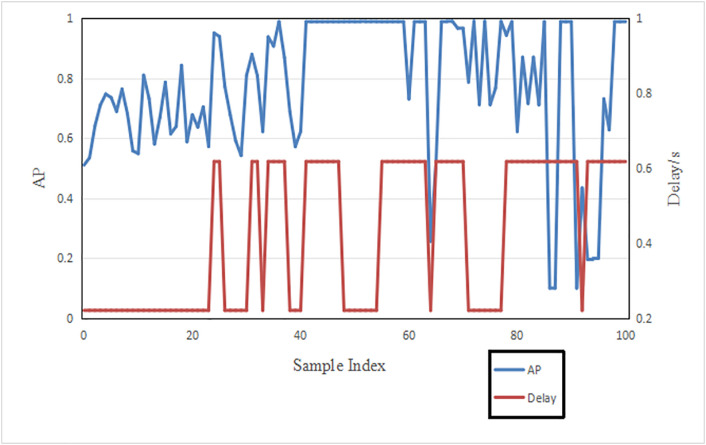
Performance under fixed point resolution and confidence thresholds.

In Section 1, the impact of point cloud resolution on system performance has been discussed in detail. As shown in Fig 9, there is a nonlinear positive correlation between delay and accuracy performance and the confidence threshold. Specifically, as shown in Fig 10, in dynamic network environments, the inference delay and accuracy under fixed bandwidth and point cloud resolution vary dynamically. Therefore, how to dynamically optimize the threshold and point cloud resolution in an unstable network environment to improve system performance is an important research issue that urgently needs to be addressed.

### 3.3. Algorithm design

#### 3.3.1. Confidence-based accuracy estimation method.

In real-time environments, obtaining the true labels of data is often challenging, making it difficult to accurately assess model performance. To address this issue, this study proposes a confidence-based accuracy estimation method, whose fundamental idea is to establish a functional mapping between confidence and accuracy using historical observation data.

**Fig 11:**
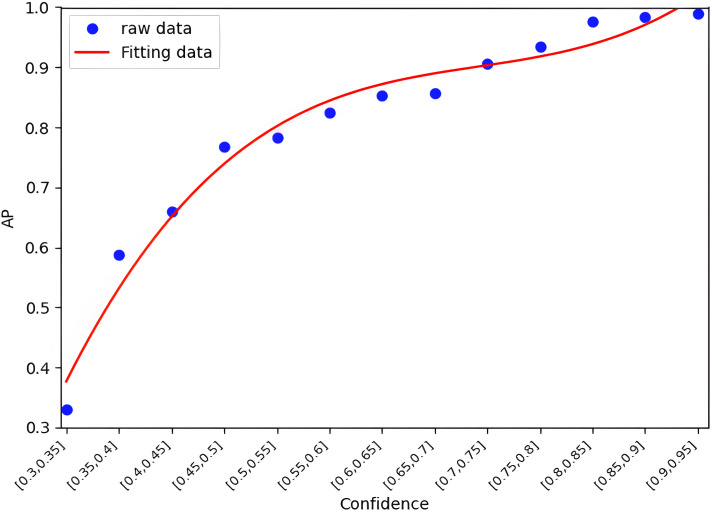
The value of terminal single-modal model (PointPillar) of the mapping function between the confidence and AP.

**Fig 12:**
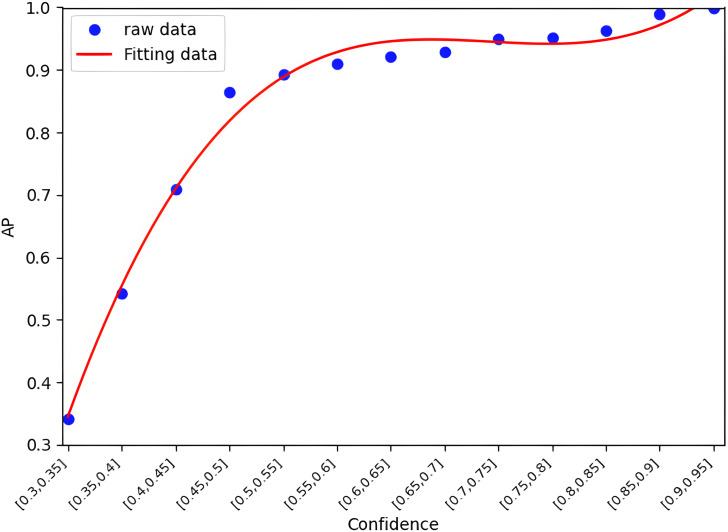
The value of the edge multimodal model (DeepInteraction) of the mapping function between the confidence and AP.

Specifically, the method uniformly partitions the confidence interval [0,1] into multiple sub-intervals. Each sample is then assigned to a corresponding interval based on its confidence value, and the average sample accuracy within each confidence interval is computed. Based on the observed distribution pattern between confidence and accuracy, a cubic function is employed for curve fitting. Fig 11 and Fig 12 illustrate the confidence–accuracy fitting relationships for the terminal single-modal model and the edge multimodal model, respectively. It can be observed that the two models exhibit different curve shapes and concavity/convexity characteristics.

On this basis, confidence–accuracy fitting functions are established for both single-modal and multimodal models, expressed mathematically as follows:


$$\overset{d}{\underset{n}{A}}=\text{5}.\text{2}\text{4}{\left(\overset{d}{\underset{n}{c}}\right)}^{\text{3}}-\text{1}\text{0}.\text{5}\text{4}{\left(\overset{d}{\underset{n}{c}}\right)}^{\text{2}}+\text{7}.\text{6}\text{6}\overset{d}{\underset{n}{c}}-\text{1}.\text{1}\text{6}$$ (5)

$$\overset{e}{\underset{n}{A}}=\text{1}\text{0}.\text{5}\text{4}{\left(\overset{e}{\underset{n}{c}}\right)}^{\text{3}}-\text{2}\text{1}.\text{9}\text{6}{\left(\overset{e}{\underset{n}{c}}\right)}^{\text{2}}+\text{1}\text{5}.\text{1}\overset{e}{\underset{n}{c}}-\text{2}.\text{5}$$ (6)

Here, $\overset{e}{\underset{n}{c}}$ and $\overset{d}{\underset{n}{A}}$ denote the confidence and estimated accuracy of sample $d_{n}$ at the terminal/edge respectively. Based on Equations (5) and (6), the accuracy of samples can be estimated in real time, providing a basis for subsequent threshold update algorithms. The proposed confidence-based accuracy estimation method is applicable to arbitrary single-modal and multimodal models.

#### 3.3.2. Dynamic threshold updating algorithm.

This paper proposes an adaptive threshold update algorithm, which primarily aims to evaluate the average accuracy of all frames from the previous cycle and, based on this evaluation, adjust the current cycle’s confidence threshold to meet the required accuracy. Specifically, when the average accuracy exceeds the required threshold, the confidence threshold is increased to reduce the offloading frequency, thereby minimizing delay. Conversely, when the average accuracy falls below the required threshold, the confidence threshold is decreased to increase the offloading frequency, thereby enhancing accuracy.

Table 4 presents the flowchart of the dynamic threshold updating algorithm, which consists of two main steps. In the first step, the mAP of the previous cycle is estimated using Equations (5) and (6). In the second step, mAP is compared with the required accuracy $A_{req}$, when mAP is larger than $A_{req}$, the threshold need to be lower, thus the update equation is shown as below, the equation (7) calculates the update threshold $r_{new}$:

**Table 4:** Algorithm 1: Dynamic threshold updating algorithm.

Dynamic threshold updating algorithm
Input: $\left(\rho_{old},\Delta \rho ,\lambda ,mAP\right)$
Output: ($\rho_{new}$)
calculate the mAP in previous time period according to equation 5 and equation 6
if $mAP<A_{req}$ do $\rho_{new}\leftarrow \rho_{old}+\lambda \cdot \Delta \rho$
if $\rho_{new}\geq \rho_{max}:$ $\rho_{new}\leftarrow \rho_{max}$ end if end if if $mAP>A_{req}$ $\rho_{new}\leftarrow \rho_{old}-\Delta \rho$ If $\rho_{new}<\rho_{min}:$ $\rho_{new}\leftarrow \rho_{min}$ end if end if return $\rho_{new}$


$$r_{new}\leftarrow r_{old}-D_{r}$$ (7)

where $D_{r}$ is the threshold increment. When the mAP is less than or equal to the required threshold $A_{req}$, the threshold is increased according to the following update formula:


$$r_{new}\leftarrow r_{old}+\lambda \times D_{r}$$ (8)

In this context, l>1 serves as a proportional factor, designed to regulate the rate of threshold increase to be greater than the rate of decrease. This design choice is driven by the fact that accuracy is treated as a primary constraint, with the fulfillment of accuracy requirements being prioritized over reducing delay and conserving network bandwidth. Additionally, the algorithm incorporates upper and lower bounds for the threshold, denoted as $r_{max}$and $r_{min}$, to prevent the occurrence of excessively high or low threshold values, which could result in either overly frequent or infrequent offloading.

#### 3.3.3. Offloading algorithm.

Based on the dynamic threshold updating algorithm, this paper designs an adaptive offloading algorithm to dynamically determine the point cloud resolution that needs to be transmitted during the offloading process. The fundamental idea is to adjust the resolution in real-time based on dynamic network bandwidth, while ensuring that the target accuracy requirements are met, thereby minimizing the overall delay as much as possible.

Algorithm 2 illustrates the flow of the adaptive offloading algorithm, as shown in the algorithm the offloading conditions are assessed explicitly based on the confidence threshold. Specifically, the confidence threshold acts as a decision gate: if the local detection confidence falls below this threshold, the task is marked for offloading to leverage the edge server’s superior accuracy; otherwise, the local result is accepted to preserve real-time performance:

First, for the current data to be detected $d_{n}$, Algorithm 2 computes the terminal confidence $\overset{d}{\underset{n}{c}}$ using a unimodal model. Second, the offloading conditions are assessed. If any of the following conditions are met, the terminal detection result is returned directly:

- $if\overset{d}{\underset{n}{c}}>r$, it indicates that the terminal detection result meets the accuracy requirements;
- $if\overset{t}{\underset{n}{t}}\left(r_{min}\right)+\overset{e}{\underset{n}{t}}\left(r_{min}\right)>T_{max},$ it indicates that the sum of the transmission and inference delays required for offloading exceeds the maximum delay constraint, meaning the network bandwidth conditions are insufficient to meet the offloading requirements, otherwise, the algorithm proceeds to the next step;
- For the data $d_{n}$to be offloaded, the algorithm iterates through the available resolution set R, electing the maximum resolution that satisfies the constraints for offloading to the edge server, and then returns the edge detection result.

The specific implementation of Algorithm 2 is shown as Table 5:

**Table 5:** Algorithm 3: Adaptive offloading algorithm.

Adaptive Offloading algorithm
Input: $\left(\overset{t}{\underset{n}{t}},\overset{e}{\underset{n}{t}},r_{max}\right)$
Output: (I)
1: I←∅
for $d_{n}$ in *D* do
Get $\overset{d}{\underset{n}{c}}$ If $\overset{d}{\underset{n}{c}}>r$ $||\overset{t}{\underset{n}{t}}\left(r_{min}\right)+\overset{e}{\underset{n}{t}}\left(r_{min}\right)>T_{max}$
return $I_{n}\leftarrow \overset{device}{\underset{n}{I}}$ else for r in R do if $\overset{t}{\underset{n}{t}}(r)+\overset{e}{\underset{n}{t}}(r)\leq T_{max}$ $I_{n}\leftarrow \overset{edge}{\underset{n}{I}}$ break end if end for end if $I\leftarrow I\cup I_{n}$ end for

## Section 4: Experimental results and analysis

### 4.1. Experimental setup

**Fig 13:**
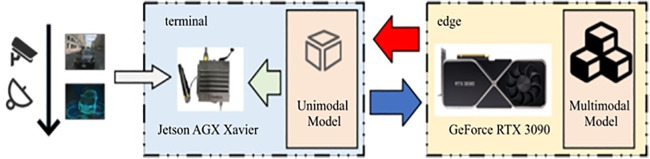
The testbed of system.

To validate the effectiveness of the proposed solution, this paper establishes an experimental testing platform for 3D object detection based on edge-assisted computation. The experiments adhere to the standard data processing protocols for 3D data as established within the OpenPCDet and MMDetection3D frameworks, encompassing methodologies such as PointPillar, DeepInteraction, BEVFusion, and TransFusion. These standardized processes ensure consistency and efficacy in data preprocessing, thereby providing a solid foundation for the research. As shown in Fig 13, the terminal device is equipped with the edge computing accelerator NVIDIA Jetson AGX Xavier, while the edge side employs the more powerful GPU server NVIDIA GeForce RTX 3090.

The comparison of the device parameters is shown in Table 6. As in Section 3, the 3D object detection models deployed on the terminal and edge sides are the single-modal model PointPillar and the multimodal model DeepInteraction, respectively. To validate the effectiveness of our proposed framework, we utilize two widely used benchmark datasets: KITTI and nuScenes, both of which provide high-precision annotations for multimodal data collected in real-world traffic environments.

**Table 6:** Comparison of equipment parameters.

Parameters	Jetson AGX	RTX 3090
Core Frequency/ MHz	854	1395
Turbo Frequency/ MHz	1377	1695
Stream Processors/ Units	512	10496
TDP Power Consumption/ W	30	350

(1) KITTI Dataset: A widely recognized benchmark in autonomous driving, capturing diverse road scenarios (urban, rural, highway) with synchronized image and LiDAR data.
(2) nuScenes Dataset: A large-scale dataset featuring 1000 scenes of 20 seconds each, collected in diverse weather and lighting conditions (e.g., rain, night) with 360-degree sensor coverage.

These datasets were selected as standard benchmarks to allow for direct comparison with state-of-the-art methods [6,34,43,49,50]. For evaluation, we use mAP as the primary metric for object detection accuracy [2,43]. Additionally, we measure computation delay to evaluate the efficiency of our proposed edge computing framework [43,50]. In this paper, a continuous sequence of 1200 frames of multimodal data from the dataset is selected as the experimental test data, primarily for detecting vehicle targets in the scene.

To evaluate the performance of the algorithm, this paper adopts the following performance metrics:

1. AP: This metric is the primary indicator used to assess the performance of object detection algorithms, and is expressed as follows:


$$AP=\int_{\text{0}}^{l}P(r)d_{r}$$ (9)

Here, P(r) is the accuracy at a threshold, r representing the overlap area between the predicted and ground truth targets, lis a value in the range [0,1], and $d_{r}$ is the length of the detection range covered by r, the AP typically provides a better reflection of the quality of object detection.

2. Delay: Defined as the total processing delay for all data divided by the total amount of data. The higher this metric, the more efficient the system’s processing capability.

Regarding algorithm comparison, this paper employs five baseline algorithms, which are described as follows:

- OD: All samples are processed for inference on the terminal device.
- AR: Resolution is adaptively adjusted under the constraint of delay, and data is offloaded to the edge server for inference. If the delay constraint is not satisfied, inference is performed on the terminal device.
- FTAR: When both a fixed confidence threshold and delay constraints are met, resolution is adaptively adjusted and offloaded to the edge server for inference. Otherwise, inference is performed on the terminal device. In this case, a fixed confidence threshold of 0.5 is set.
- OB [51]: The target accuracy is set according to the accuracy of the object being detected, and the PSO algorithm is used to select the highest resolution that satisfies both delay and accuracy constraints for offloading to the edge server.
- VA [52]: A cost function is established based on resolution and accuracy. A greedy algorithm is used to traverse selectable resolutions, finding the minimum resolution that satisfies the target accuracy, which is then offloaded to the edge server.

**Fig 14:**
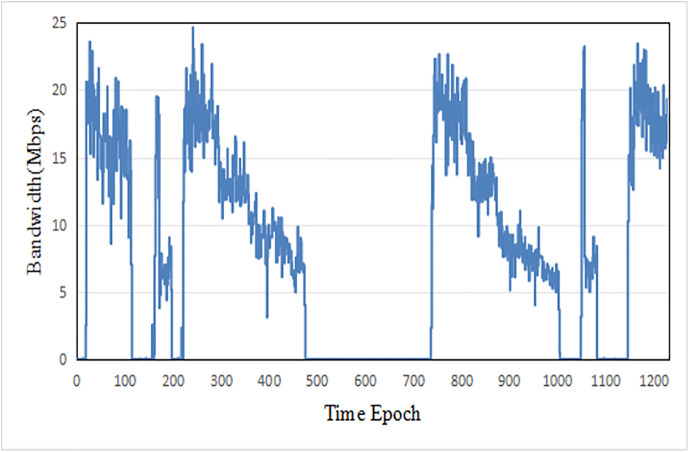
Transmission rate variation under different time epochs.

In terms of parameter settings, this paper tests the performance under various accuracy requirements with different confidence increments $D_{r}$, the testing environment is a dynamic network, and the bandwidth fluctuation curve is shown in Fig 14.

**Fig 15:**
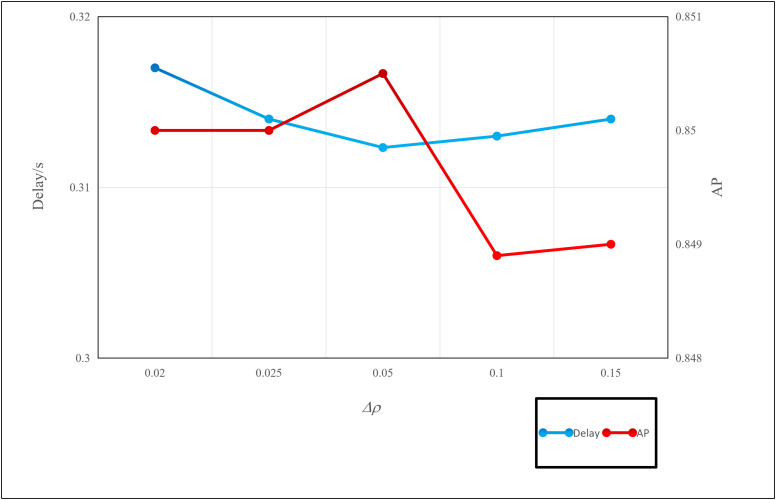
Performance under different values ofΔρ.

We set the values of Δρacross six different ranges, and test the delays and accuracies of our algorithm. As shown in Fig 15, algorithm achieves the highest accuracy and lowest average delay when Δρ=0.05. Thus, we set Δρ=0.05 in the subsequent experiments. Additionally, the scale factor λ is set to 3 based on empirical testing. This value provides an optimal balance between responsiveness to new channel conditions and robustness against transient noise. A higher λ makes the system too volatile, while a lower λ causes lag in response. Unless specifically stated, all experiments are conducted with the default parameter configuration.

### 4.2. Performance comparisons

#### 4.2.1. Performances of different algorithms.

**Fig 16:**
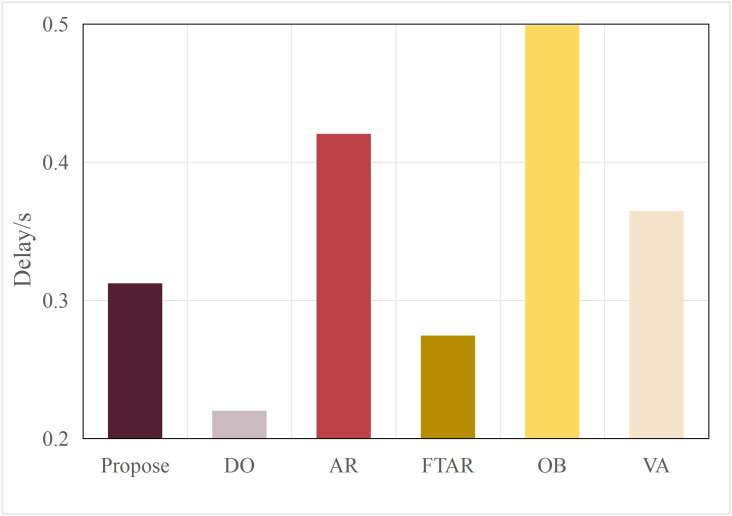
Delay values of different algorithms.

**Fig 17:**
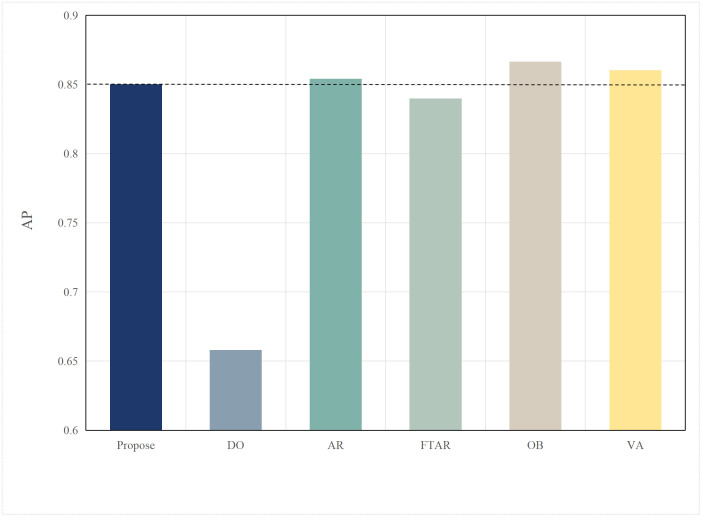
AP values of different algorithms.

This paper tests and compares the performance of various offloading algorithms under $A_{req}=\text{0}.\text{8}\text{5}$, with the experimental results shown in  Fig 16 and 17.

In terms of delay, the OD algorithm achieved the minimum value because it only performs inference locally. On the other hand, OB experienced the maximum delay, as the PSO algorithm requires multiple iterations to select the optimal resolution. The VA algorithm employs a traversal method for resolution selection, which introduces additional delay during the traversal process. The AR algorithm also had a longer delay, as it only considers resolution adaptation, leading to frequent offloading. FTAR, which accounts for a fixed confidence threshold and resolution adaptation, reduces the amount of offloaded data compared to AR, resulting in a smaller delay.

In terms of accuracy, OB achieved the best AP value due to the finer search granularity provided by the particle swarm iterations, enabling it to find the maximum resolution that meets the delay constraint. VA, however, uses a coarser traversal granularity compared to OB, resulting in a slightly lower accuracy. Meanwhile, OD, which performs inference locally, resulted in an AP value lower than the required accuracy of 0.85. The proposed algorithm in this paper, which combines dynamic threshold tuning and resolution adaptation, minimizes delay while meeting the accuracy requirement, achieving a better balance compared to the benchmark algorithms.

#### 4.2.2. Performances of different edge inference models.

To verify the scalability of the algorithm, this paper replaces the multimodal models at the edge with BEVFusion and TransFusion. As shown in Fig 5 and Fig 6, compared to DeepInteraction, the AP values of BEVFusion and TransFusion have significantly decreased. If the target $A_{req}$is still maintained at 0.85, it will lead to frequent offloading, resulting in severe delays. Therefore, in this set of tests, the paper adjusts $A_{req}$to 0.8.

**Fig 18:**
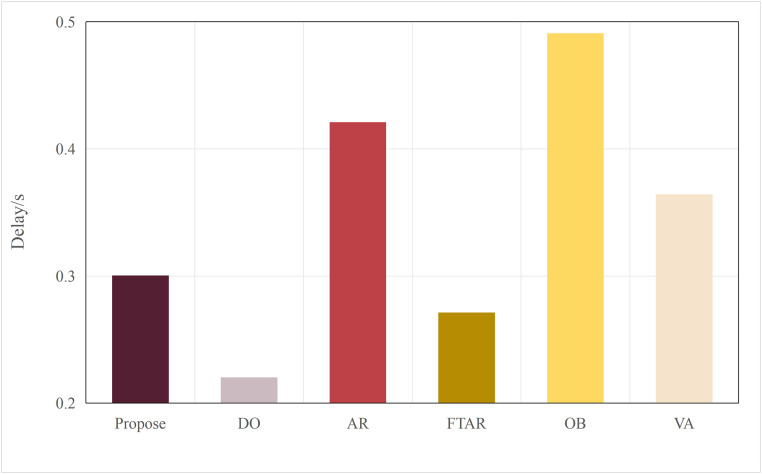
Delay values of different algorithms(BEVFusion used for edge inference).

**Fig 19:**
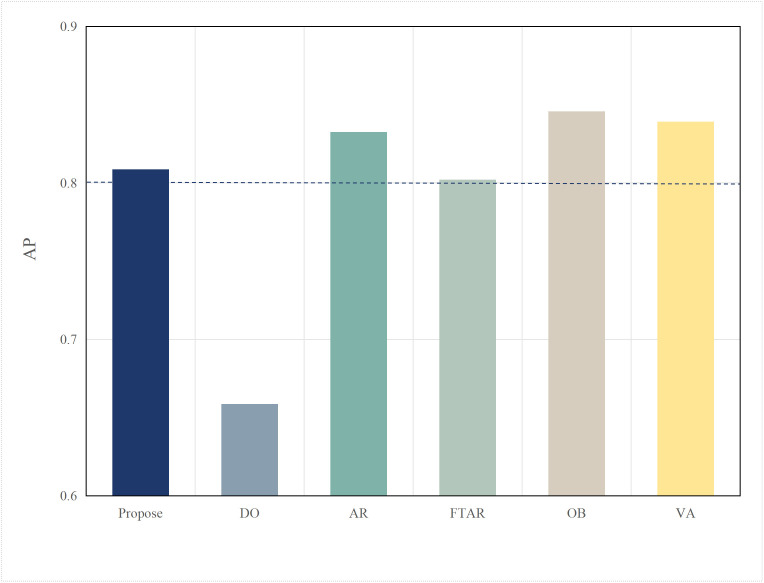
AP values of different algorithms(BEVFusion used for edge inference).

Fig 18 and Fig 19 show the performance when the edge model is switched to BEVFusion. Compared to  Fig 16 and 17, except for the OD algorithm, the AP values of the other algorithms have decreased to varying degrees. The OD algorithm, which does not perform task offloading, maintains both accuracy and delay at their lowest and most stable values. AR, OB, and VR still achieve relatively high AP performance but at the cost of higher delays. Notably, due to the lower accuracy requirement, the AP value of FTAR just meets the $A_{req}=\text{0}.\text{8}$ achieving the lowest delay among the algorithms that meet the delay requirements. The proposed algorithm in this paper, by adjusting according to the target accuracy, also adjusts the offloading strategy in a timely manner. While meeting the $A_{req}$, it achieves the second-best delay performance among the algorithms that meet the conditions.

**Fig 20:**
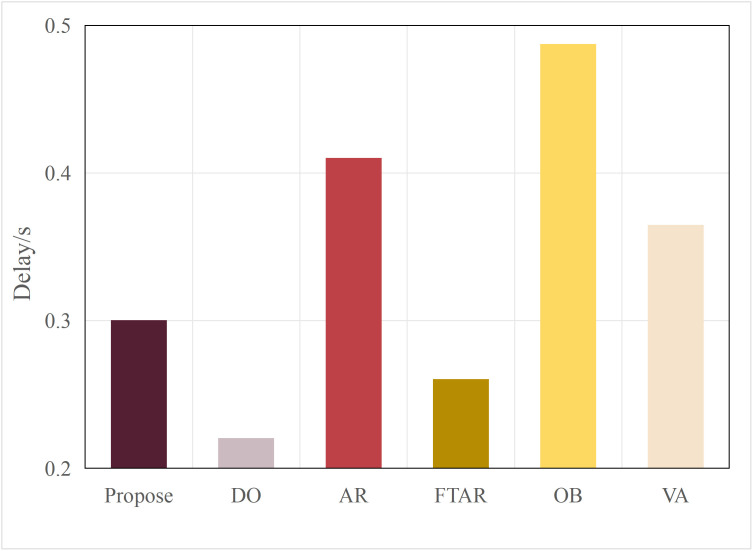
Delay values of different algorithms(TransFusion used for edge inference).

**Fig 21:**
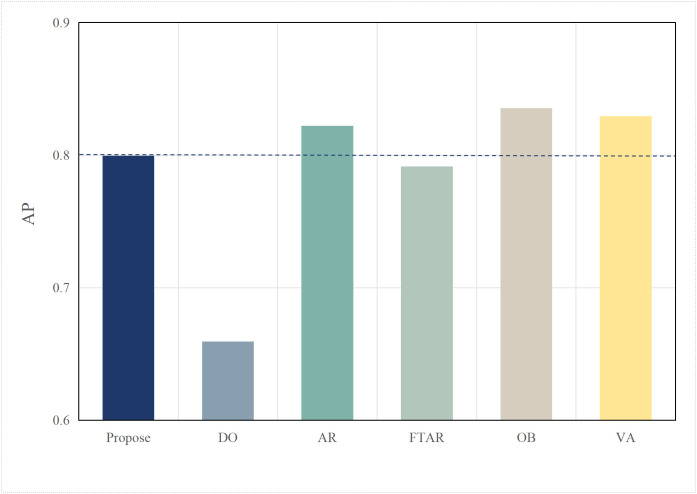
AP values of different algorithms(TransFusion used for edge inference).

Fig 20 and Fig 21 show the performance when the edge model is switched to TransFusion. TransFusion’s detection accuracy and delay are both lower than those of BEVFusion. Therefore, when the edge model is TransFusion, the overall AP value decreases compared to when the edge model is BEVFusion. Notably, the FTAR algorithm cannot meet the $A_{req}=\text{0}.\text{8}$. However, the proposed algorithm adjusts the offloading frequency adaptively by tuning the confidence threshold and resolution, still satisfying the 0.8 accuracy requirement, while achieving the lowest delay performance among the algorithms that meet the accuracy requirement. The above analysis shows that the proposed algorithm has a certain degree of generalization ability across different edge models.

The proposed algorithm achieves delays of 0.42s and 0.35s when using BEVFusion_L and TransFusion_L as terminal models, both of which are higher than the delay of PointPillar as the terminal model (approximately 0.31s, as shown in Fig 16 and Fig 17. This is because both BEVFusion_L and TransFusion_L have longer inference delays compared to PointPillar (as shown in Fig 7 and Fig 8). However, when BEVFusion_L and TransFusion_L are used as the terminal models, there is no significant advantage in AP values over PointPillar. This is because the proposed edge-terminal collaboration framework effectively compensates for the insufficient inference accuracy of the terminal models on complex samples through the edge model.

**Fig 22:**
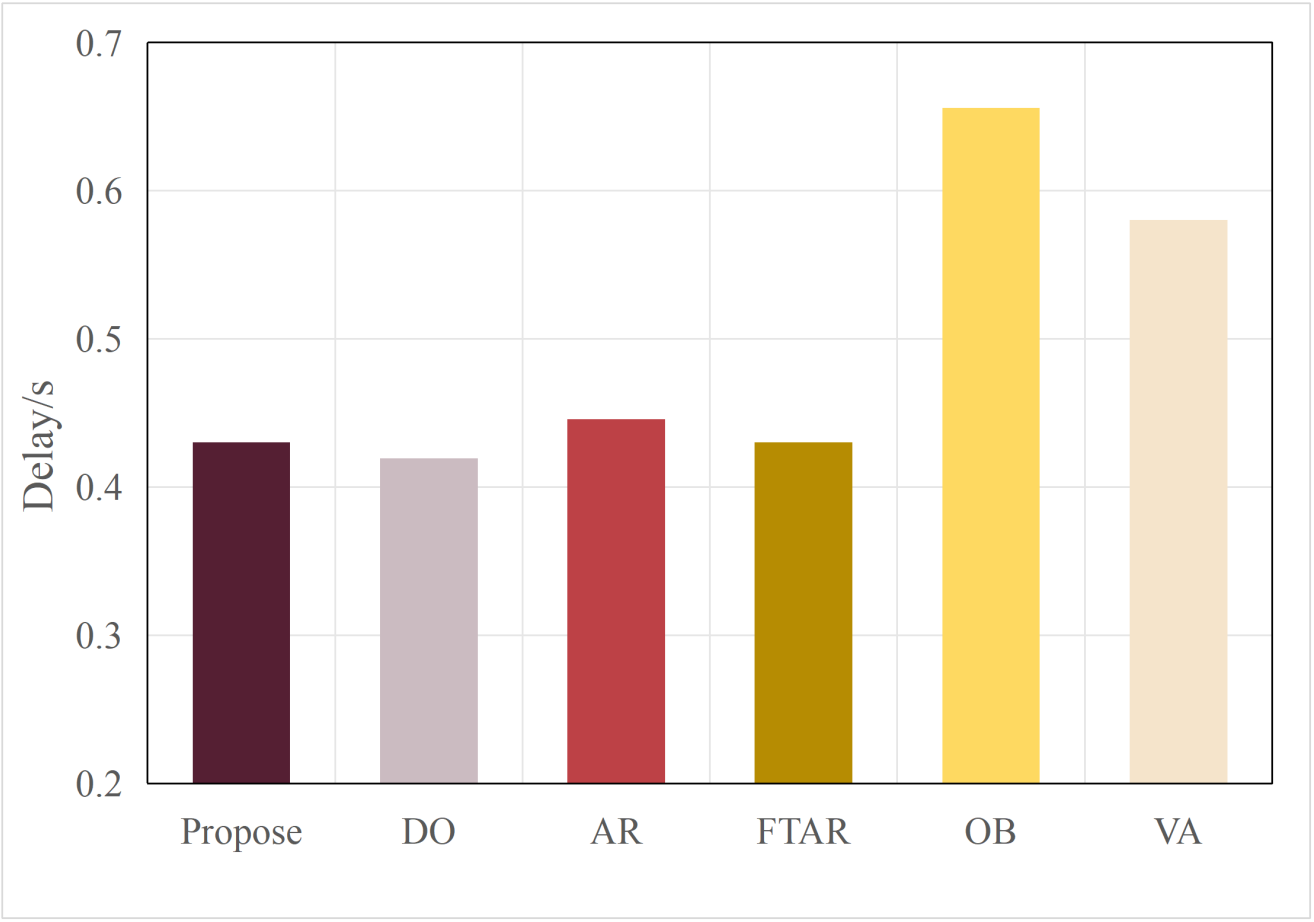
Delay values of different algorithms(BEVFusion_L used for device inference).

**Fig 23:**
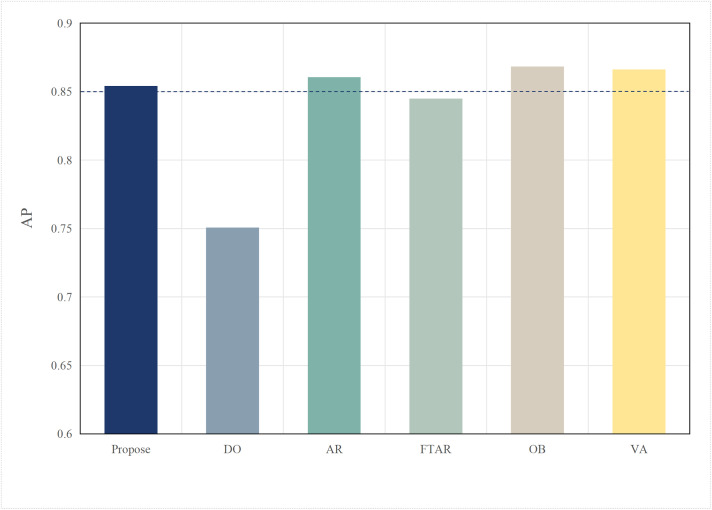
AP values of different algorithms(BEVFusion_L used for device inference).

**Fig 24:**
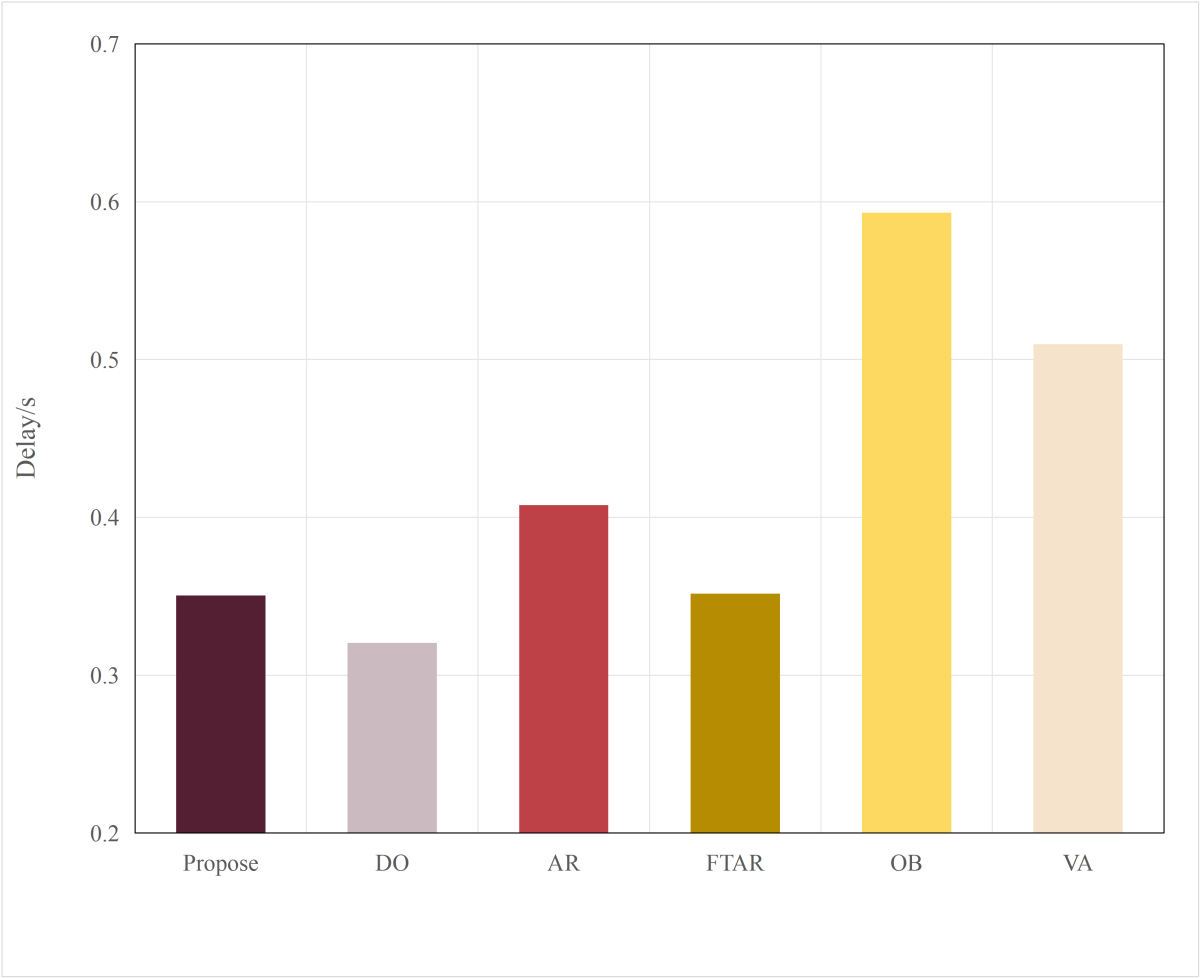
Delay values of different algorithms(TransFusion_L used for device inference).

**Fig 25:**
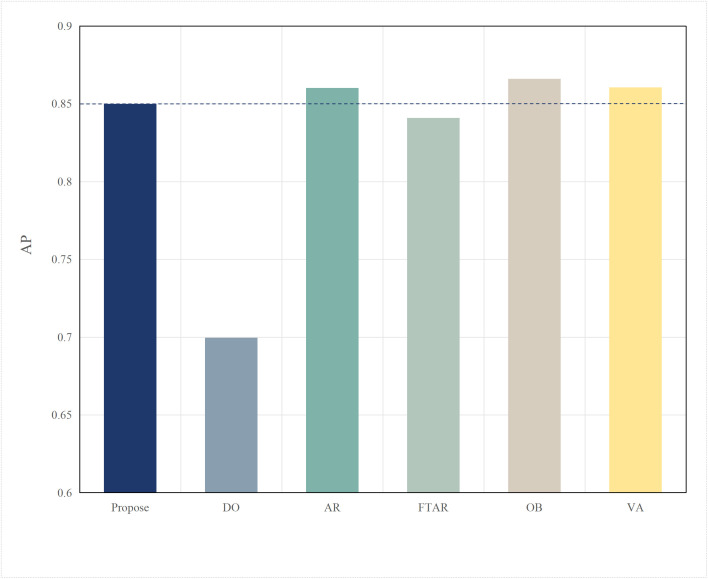
AP values of different algorithms(TransFusion_L used for device inference).

Additionally, as shown in Figs 22–25, compared to other methods, the proposed offloading algorithm achieves the lowest delay while meeting the accuracy requirement, achieving the best balance. This further validates the generalization ability of the proposed algorithm across different terminal model scenarios.

#### 4.2.3. Performances of different datasets.

**Fig 26:**
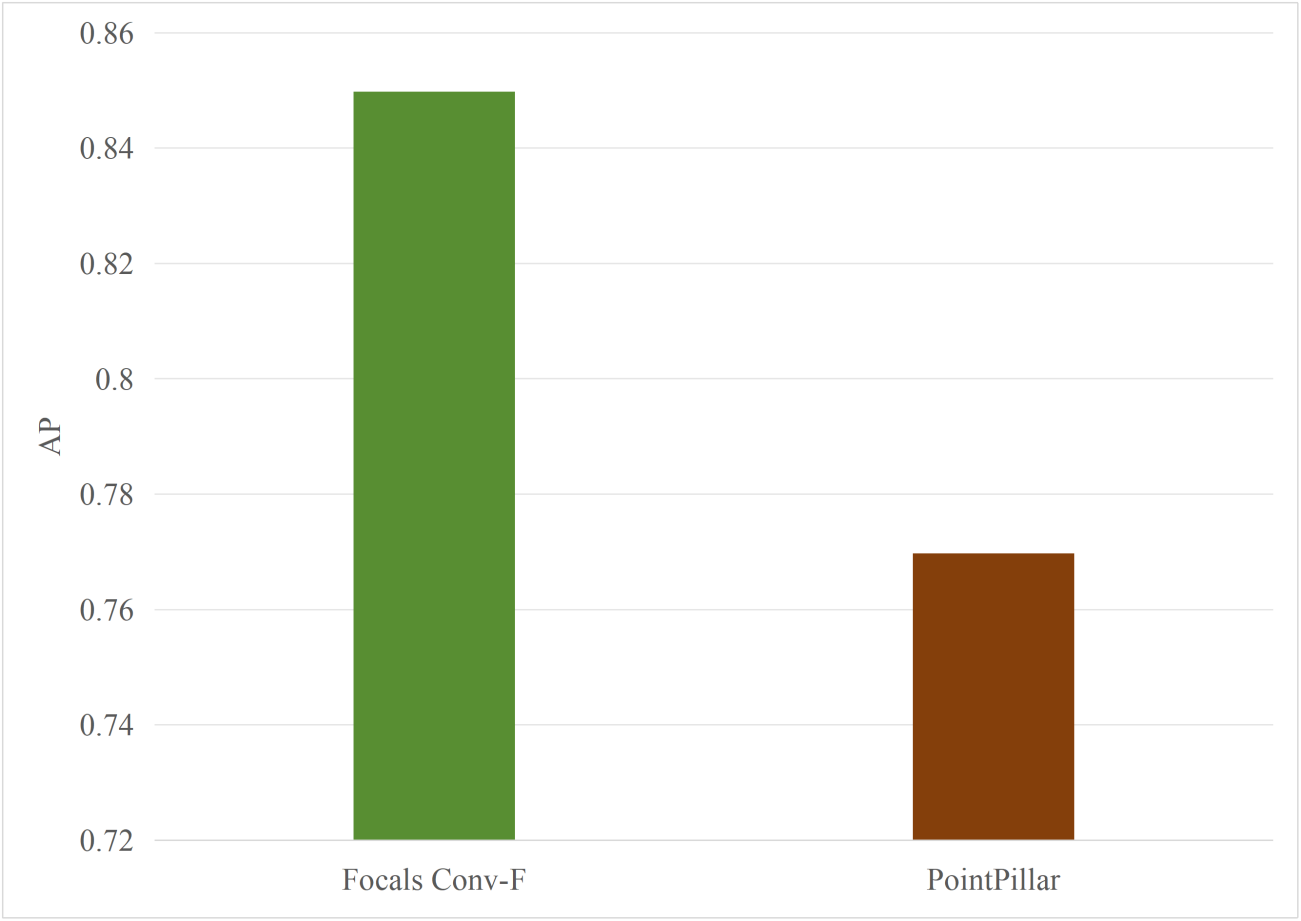
Comparison of AP.

In this section, algorithm performance tests were conducted on the autonomous driving evaluation dataset KITTI to verify the generalization ability of the algorithm across different datasets. Compared to nuScenes, the KITTI dataset has a more limited perspective. Specifically, nuScenes image data is captured from six different viewpoints, while the point cloud has a 360-degree field of view and a large range. In contrast, KITTI image data is captured from a single viewpoint, and the point cloud has a 180-degree field of view with a smaller range. The data formats of the nuScenes and KITTI datasets are inconsistent, which makes the DeepInteraction multimodal model, originally deployed on the edge, incompatible. Therefore, the edge model was replaced with the Focals Conv-F [53] model, which performs one of the best on the KITTI dataset, while the terminal model continued using the PointPillar model. The accuracy comparison of these two models on the KITTI dataset is shown in Fig 26. As shown in the Fig 26, due to the single viewpoint of KITTI’s image and point cloud data, the data is not as rich, and the Focals multimodal model achieves an accuracy of about 0.85 on the KITTI dataset. Compared to the nuScenes dataset, this represents a significant decrease in accuracy. Therefore, in this section, the target accuracy was adjusted downward to 0.8.

**Fig 27:**
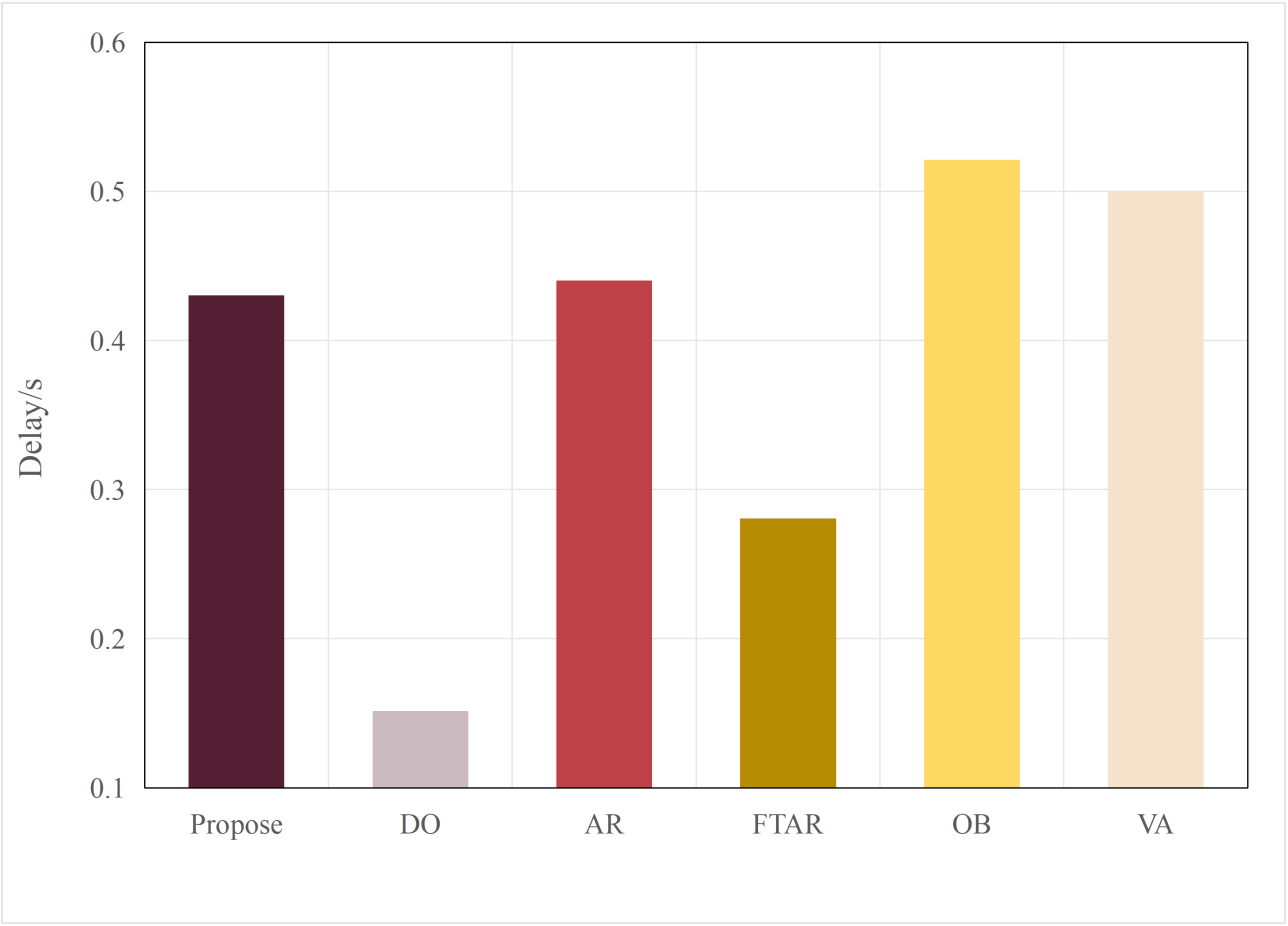
Delay values of different datasets(KITTI).

**Fig 28:**
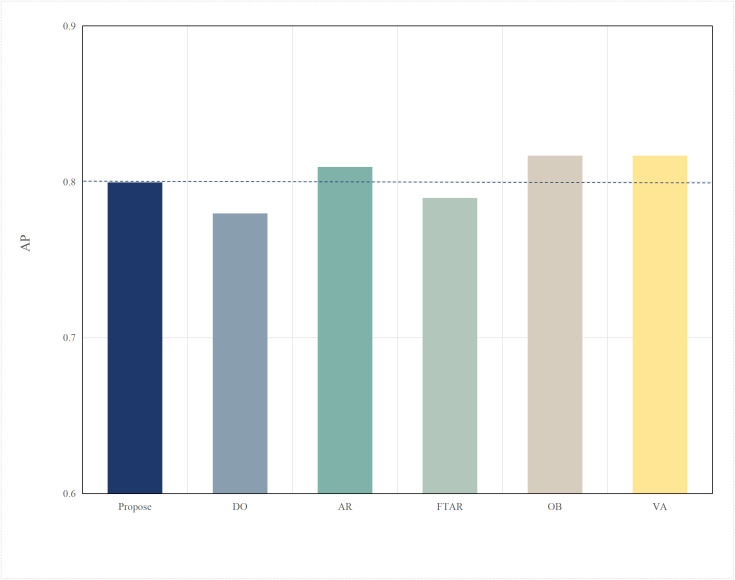
AP values of different datasets(KITTI).

The performance comparison results of different algorithms on the KITTI dataset are shown in Fig 27 and Fig 28. Similar to their performance on the nuScenes dataset, the FR algorithm, due to the use of a fixed original resolution, results in an excessively large data volume, which cannot meet the delay threshold set for offloading. As a result, the inference task can only be performed on the terminal. Therefore, the performance of FR is similar to that of the DO algorithm, with the lowest accuracy and the lowest delay. Although the FTAR algorithm can adaptively adjust the resolution, it has a fixed confidence threshold, which prevents it from filtering offloaded data. Its accuracy, compared to OD, does not show significant improvement. The AR algorithm adjusts the resolution adaptively to increase the likelihood of offloading and improve accuracy (about 0.81), but the large amount of offloaded data leads to higher delay. In the OB algorithm, the multiple iterations of the particle swarm optimization (PSO) result in the highest delay (about 0.52s), while it also favors higher resolutions, leading to the highest accuracy, approximately 0.82. The VA algorithm finds the minimum resolution that satisfies the target accuracy, resulting in a slightly lower delay than OB. The algorithm proposed in this paper maintains the most balanced performance, achieving the lowest delay (about 0.43s) while meeting the accuracy requirements.

#### 4.2.4. Performances of different point cloud compression algorithms.

**Fig 29:**
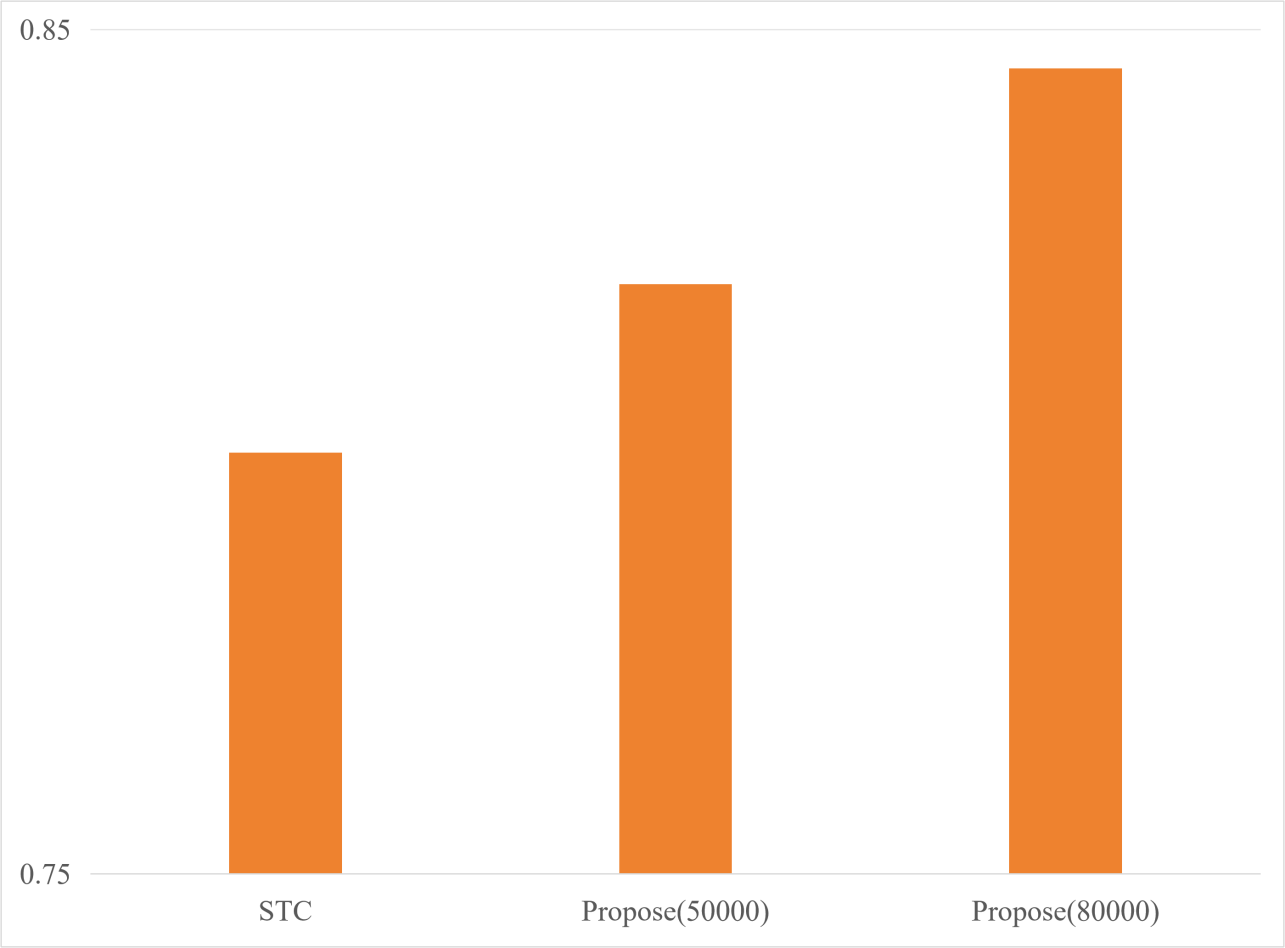
Performance of different point cloud compression algorithms.

In this section, the proposed point cloud resolution compression algorithm is compared with a real-time spatial compression algorithm, STC [54], in terms of performance. STC removes redundant information from point cloud data by combining spatial and temporal encoding to reduce the size of the point cloud data. This study selected 1200 frames of point cloud data from the KITTI dataset, applying different compression algorithms and comparing various metrics. As shown in Table 5, during the compression phase, the STC algorithm has a larger delay due to its complex computation process, while the delay of the proposed point cloud compression strategy is negligible. In terms of compression effectiveness, as shown in Table 5, the proposed compression algorithm generates smaller data sizes compared to STC. Specifically, even when the point cloud resolution is set to 80,000, the data size obtained by the proposed method is still smaller than that of the STC method. Moreover, as shown in Fig 29, the proposed method achieves better accuracy during terminal model inference with a smaller data size.

#### 4.2.5. Performances of different bandwidth.

**Fig 30:**
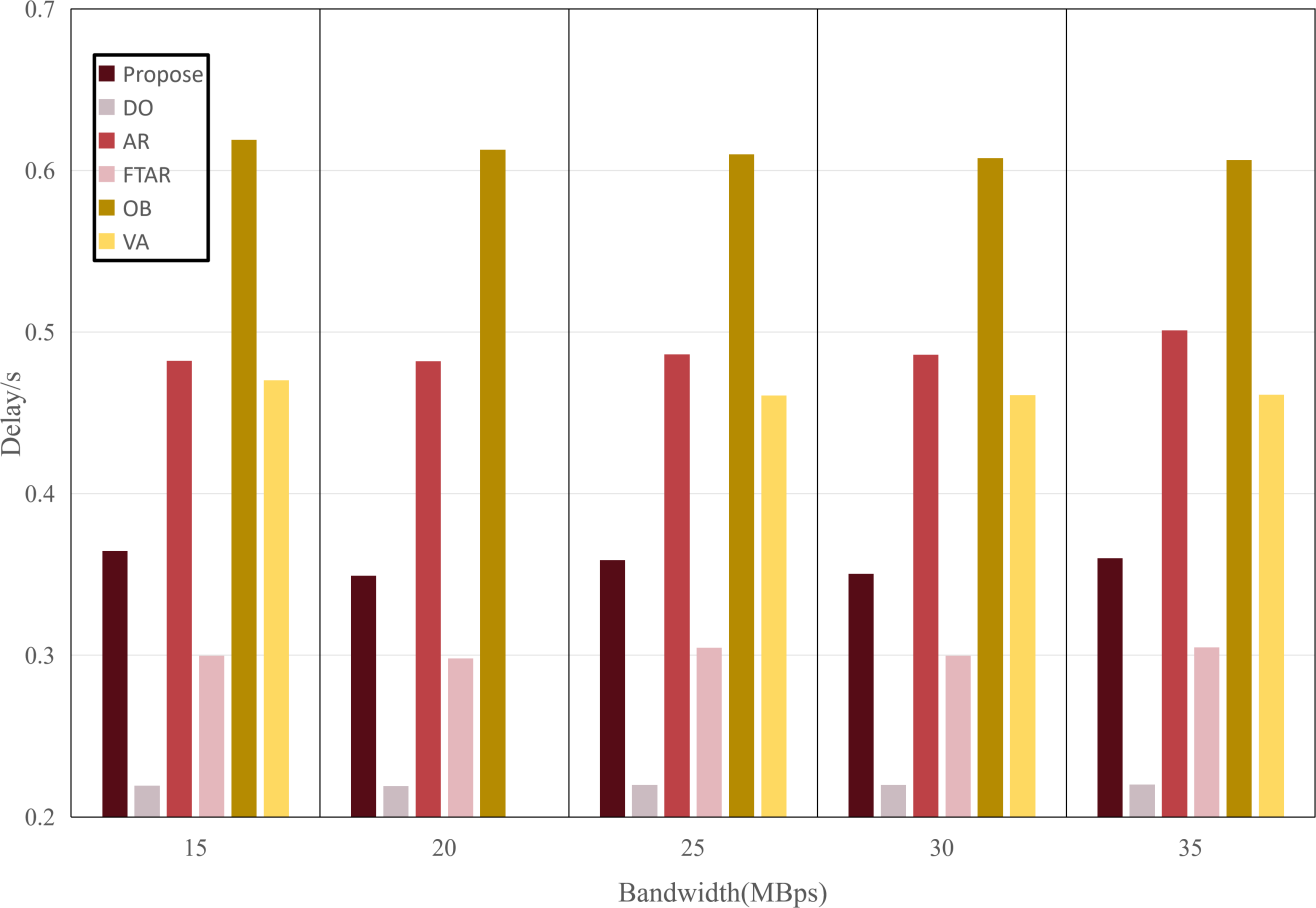
Delay values of different bandwidths.

**Fig 31:**
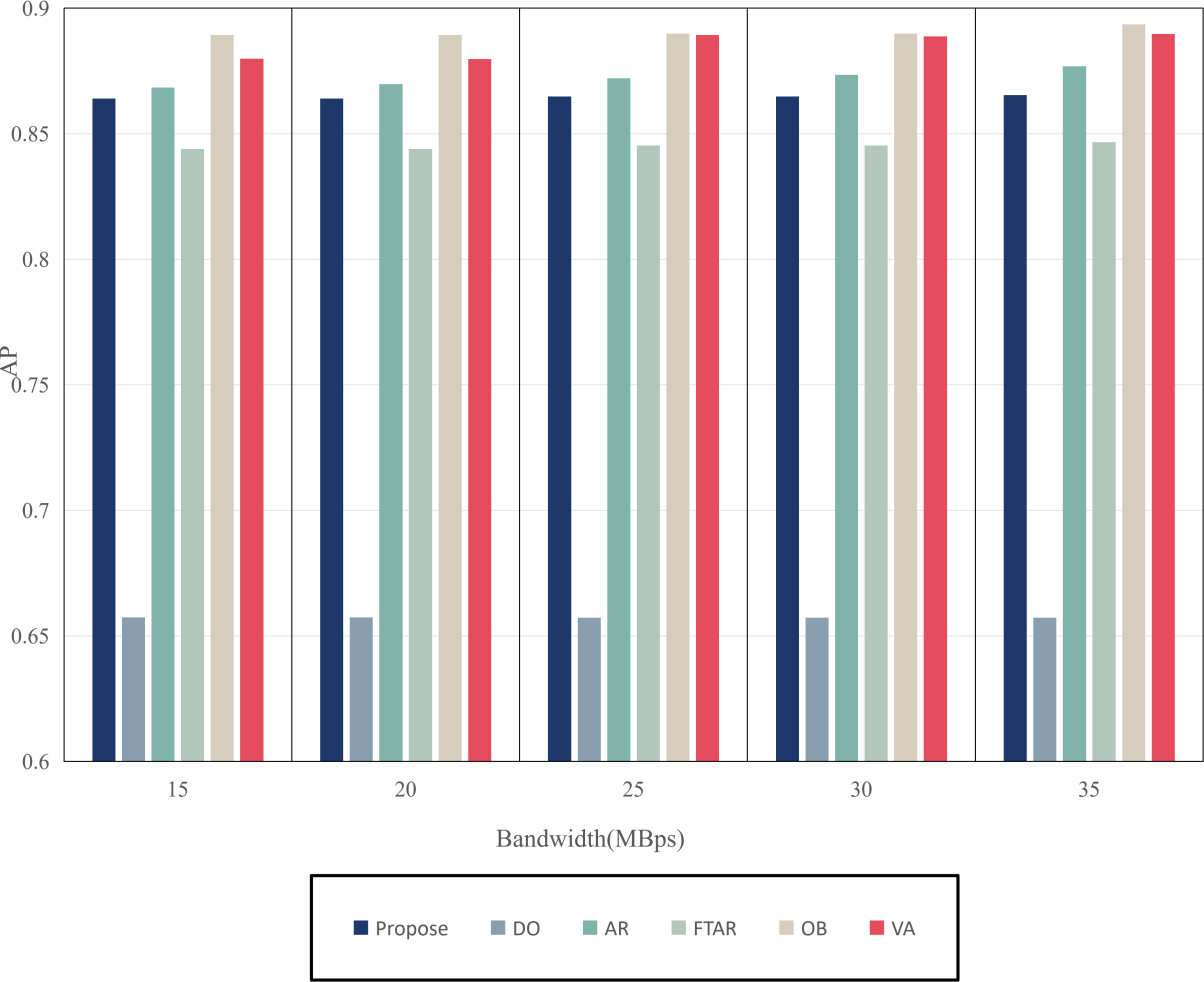
AP values of different bandwidths.

To verify the adaptability of the algorithm, the performance was tested under different network environments. The experimental results are shown in Fig 30 and Fig 31. The OD algorithm, due to its lack of offloading, is unaffected by the network environment, maintaining the lowest accuracy and delay without fluctuations. As the transmission rate increases, the resolution offloaded by the AR algorithm also increases, leading to higher delay and accuracy. Similarly, FTAR increases its offloading resolution to improve accuracy as the transmission rate rises, but still fails to meet the target accuracy requirements. Both OB and VA achieve optimal accuracy, but their delay costs are the highest. In contrast, the proposed algorithm maintains the target accuracy while achieving the lowest delay across all network conditions, thus balancing both accuracy and delay.

## Section 5: Conclusions

In the field of autonomous driving, 3D object detection has high requirements for both accuracy and real-time performance. Currently, relying solely on the limited computational capacity of on-board terminals cannot meet these demands. To address this challenge, this paper proposes a novel edge-assisted 3D object detection framework that leverages the heterogeneous computational resources of terminal devices and edge servers to achieve collaborative inference between single-modal and multimodal models. Based on this, the paper analyzes in detail the impact of point cloud resolution and confidence thresholds on delay and accuracy, providing decision guidance for offloading inference tasks based on these two metrics. Specifically, a mixed-integer programming model with point cloud resolution and confidence threshold as optimization variables is established, aiming to minimize delay while meeting accuracy requirements. To assess accuracy in real-time, a confidence-accuracy function mapping relationship is established based on historical confidence-accuracy distribution patterns and cubic fitting functions. In order to adapt to dynamic network environments, a dynamic threshold update algorithm is proposed, which adjusts the threshold by periodically evaluating accuracy. Additionally, an adaptive decision algorithm is designed, which real-time adjusts point cloud resolution to achieve a balance between delay and accuracy. Finally, an edge-terminal collaborative hardware experimental platform is built, and the proposed solution is validated on real autonomous driving datasets, nuScenes and KITTI. Experimental results show that, under various testing scenarios, including different network bandwidths and model deployments, the proposed solution achieves a more comprehensive performance in terms of both delay and accuracy compared to the baseline solutions.

However, this study has not yet fully addressed the feasibility of real-time implementation in hardware-constrained systems, accuracy in small object detection, and performance under adverse weather conditions. Future research will focus on assessing the applicability of this algorithm in real-time systems, particularly for autonomous driving and robotics. This will involve exploring advanced calibration techniques to enhance model confidence, which is vital for systems where reliability is of extreme importance. Additionally, we plan to address the specific challenges of insufficient feature representation and background interference in small object detection, and evaluate the system’s robustness under adverse weather conditions, such as rain or fog, to ensure consistent performance in dynamic environments. Furthermore, to overcome the limitations of optical sensors in adverse weather conditions, future research will explore the integration of wireless sensing modalities. Recent advancements in 6G Integrated Sensing and Communication (ISAC) [55] have shown that wireless signals (e.g., 6.75 GHz) can generate high-fidelity point clouds via Channel State Information (CSI). Fusing these wireless-generated point clouds with visual and LiDAR data could drastically improve system robustness and safety in all-weather autonomous driving scenarios. In this work, network parameters (e.g., available bandwidth and transmission delay) are empirically measured on real hardware platforms under actual network conditions, inherently accounting for factors like path loss, multipath fading, and interference. Future research will extend these measurements to high-mobility scenarios through field tests at various device velocities, incorporating Doppler effect modeling to analyze the impact of mobility on transmission reliability, offloading latency, and detection accuracy. This will facilitate the development of mobility-aware adaptive offloading strategies for dynamic edge-device collaboration.

## Abbreviations

AP: Average Precision
CAVs: Connected and Autonomous Vehicles
C-V2X: Cellular Vehicle-to-Everything
FTAR: Fixed Threshold Adaption Resolution
IoT: Internet of Things
mAP: Average Mean Precision
MEC: Multi-access Edge Computing
OB: Object-Based resolution selection
OD: Only Device
PSO: Particle Swarm Optimization
STC: Spatio-Temporal Lidar Point Cloud Compression
VA: dynamic adaptive offloading for Video Analytics
V2I: Vehicle-to-Infrustructure
V2X: Vehicle-to-Everything.
